# Microbiota and Resveratrol: How Are They Linked to Osteoporosis?

**DOI:** 10.3390/cells13131145

**Published:** 2024-07-03

**Authors:** Christine Meyer, Aranka Brockmueller, Vicenç Ruiz de Porras, Mehdi Shakibaei

**Affiliations:** 1 Chair of Vegetative Anatomy, Institute of Anatomy, Faculty of Medicine, Ludwig-Maximilians-University Munich, Pettenkoferstr. 11, D-80336 Munich, Germany; christine.meyer@med.uni-muenchen.de (C.M.); aranka.brockmueller@med.uni-muenchen.de (A.B.); 2 CARE Program, Germans Trias i Pujol Research Institute (IGTP), Camí de les Escoles, s/n, Badalona, 08916 Barcelona, Spain; vruiz@igtp.cat; 3 Badalona Applied Research Group in Oncology (B⋅ARGO), Catalan Institute of Oncology, Camí de les Escoles, s/n, Badalona, 08916 Barcelona, Spain; 4 GRET and Toxicology Unit, Department of Pharmacology, Toxicology and Therapeutic Chemistry, Faculty of Pharmacy and Food Sciences, University of Barcelona, 08028 Barcelona, Spain

**Keywords:** resveratrol, microbiome, dysbiosis, osteoporosis, gut–bone axis, prebiotics, epigenetics, bone metabolism

## Abstract

Osteoporosis (OP), which is characterized by a decrease in bone density and increased susceptibility to fractures, is closely linked to the gut microbiota (GM). It is increasingly realized that the GM plays a key role in the maintenance of the functioning of multiple organs, including bone, by producing bioactive metabolites such as short-chain fatty acids (SCFA). Consequently, imbalances in the GM, referred to as dysbiosis, have been identified with a significant reduction in beneficial metabolites, such as decreased SCFA associated with increased chronic inflammatory processes, including the activation of NF-κB at the epigenetic level, which is recognized as the main cause of many chronic diseases, including OP. Furthermore, regular or long-term medications such as antibiotics and many non-antibiotics such as proton pump inhibitors, chemotherapy, and NSAIDs, have been found to contribute to the development of dysbiosis, highlighting an urgent need for new treatment approaches. A promising preventive and adjuvant approach is to combat dysbiosis with natural polyphenols such as resveratrol, which have prebiotic functions and ensure an optimal microenvironment for beneficial GM. Resveratrol offers a range of benefits, including anti-inflammatory, anti-oxidant, analgesic, and prebiotic effects. In particular, the GM has been shown to convert resveratrol, into highly metabolically active molecules with even more potent beneficial properties, supporting a synergistic polyphenol–GM axis. This review addresses the question of how the GM can enhance the effects of resveratrol and how resveratrol, as an epigenetic modulator, can promote the growth and diversity of beneficial GM, thus providing important insights for the prevention and co-treatment of OP.

## 1. Introduction

The human gut microbiota (GM) is estimated to consist of more than 100 trillion microbes with approximately 35,000 bacterial species living in natural symbiosis with the entire human organism [1]. In recent years, the interactions between the GM and various health conditions have increased significantly, particularly its critical role in regulating bone metabolism and the development of osteoporosis (OP) [2]. The interaction or crosstalk between GM and bone tissue is referred to as the gut–bone axis [3], which is mediated by several mechanisms, such as the GM-derived expression of vitamins and bioactive metabolites such as anti-inflammatory short-chain fatty acids (SCFA), which have been found to modulate major inflammatory signaling pathways such as nuclear factor-kappa B (NF-κB) at the epigenetic level [4]. Additionally, the GM has been found to interact with vitamin D [5] and several hormones, including estrogen [6], thereby fulfilling an essential function for a healthy, balanced hormonal status. Considering that GM and their metabolites are further critical contributors to a physiological immune response through modulation of immunoglobulin A production [7] and regulatory T cell (Treg) activity [8], alterations in GM diversity and composition in conjunction with altered GM-derived metabolites are postulated to be the origin for a variety of chronic inflammatory diseases, which consequently require GM targeting as part of a preventive or co-treatment regimen [9]. In this regard, imbalances in the GM, also referred to as dysbiosis, have been identified with a significant reduction in beneficial GM-derived metabolites, such as decreased levels of SCFA and increased harmful GM-derived metabolites such as Trimethylamine N-oxide (TMAO), both contributing to increased local and systemic inflammatory processes, with the activation of NF-κB [9]. This has been shown to interfere with processes such as bone remodeling, leading to increased osteoclastogenesis and decreased osteogenesis [9]. 

In this context, numerous individual lifestyle factors such as dietary habits [10], physical activity [11], long-term psychological stressors [12], dysregulated circadian rhythm [13], and consumption of harmful stimulants [14] have been demonstrated in association with the development of dysbiosis. These factors are widely considered to be risk factors for many chronic inflammatory diseases, including OP [15]. Interestingly, long-term or repeated use of medications, for example, nonsteroidal anti-inflammatory drugs (NSAIDs) [16], antibiotics [17], proton pump inhibitors (PPIs) [18], and chemotherapeutic agents [19], also effect GM composition significantly by suppressing beneficial GM. In addition, endogenous risk factors such as estrogen deficiency are associated with altered GM, including the loss of beneficial GM species [20] and a higher abundance of harmful GM, leading to increased intestinal permeability, systemic inflammation, and dysregulation of bone remodeling [21]. 

In view of the fact that OP often remains undiagnosed for a long time, even after typical osteoporotic fractures [22], an early preventive treatment of an underlying dysbiosis is postulated to counteract the processes of osteoporotic bone loss at an early stage. In fact, there is already clinical evidence that the modulation of dysbiosis in OP patients positively correlates with improved bone regeneration [23]. However, many conventional standard therapies for OP are mainly based on drug treatment with monoclonal antibodies against receptor activator of NF-κB ligand (RANKL), which act as a single-target molecule, reducing bone resorption but are not able to actively stimulate the expression of necessary osteogenic transcription factors such as Runt-related transcription factor 2 (Runx2) and Osterix [24,25]. Therefore, the education and further development of new adjunctive patient-centered therapeutic regimes are highly required. 

One promising approach is the preventive and adjunct use of natural phytopharmaceuticals such as polyphenols, which act prebiotically by promoting growth and diversity of beneficial GM while providing multiple additional features including the ability to stimulate intrinsic tissue regeneration by modulating pro-inflammatory signaling pathways such as NF-*κ*B [26]. Currently, resveratrol is one of the best-studied natural polyphenols, which is found in numerous foods, including fruits, vegetables, nuts, seeds, and herbs [27,28]. Interestingly, resveratrol has a proven bone regeneration effect due to its phytoestrogenic nature, which is an important preventative attribute for OP, particularly for women going through menopause or individuals recovering from hormonal therapies for conditions such as breast cancer [15] or prostate cancer [29]. Interestingly, GM has been demonstrated to metabolize resveratrol into highly biologically active metabolites with even more potent anti-inflammatory and anti-cancer effects than resveratrol itself, supporting the beneficial multifunction of this phytopharmaceutical [30]. Resveratrol’s prebiotic and anti-inflammatory function has been demonstrated to contribute to the beneficial diversity of the GM by modulating the gut microenvironment and thereby strengthening the intestinal mucosal barrier [26,31]. Therefore, it is suggested that resveratrol primarily targets the gut microenvironment, including modulation of GM, thereby stimulating GM-derived metabolites such as SCFA [32] and also potent GM-derived resveratrol-metabolites, which might explain the numerous clinical benefits although resveratrol’s oral bioavailability after metabolism in the gut and liver is only 1% [27].

In this review, we discuss the multifunctionality of resveratrol in terms of prebiotic function, and stimulation of intestinal mucosal regeneration, both leading to modulation of GM and associated gut–bone axis. In addition, we address the recognized phytoestrogenic effects of resveratrol at the epigenetic level in terms of bone regeneration, supporting the clinical use of resveratrol as a preventive and adjuvant natural compound in patients with both primary and secondary OP.

## 2. Osteoporosis

OP is classified as a systemic chronic inflammatory disease that is characterized by massive loss of bone mass, leading to increased bone fragility and risk of osteoporotic fractures [33,34]. The commonly quoted incidence of OP of around 200 million people worldwide is hypothesized much higher, as the incidence of the primary OP, as well as underlying primary diseases of secondary OP itself, is much higher and expected to continue to rise sharply in the near future due to increasing life expectancy and the associated inflammatory aging process [15,22]. The insidious fact about OP is that dysregulated bone remodeling processes in favor of increased osteoclastogenesis often remain undetected for a long time, even after typical fracture sites such as the femoral-hip or lumbar spine [22]. Currently, OP can be treated with bisphosphonates and mono-target antibodies, but this is associated with various side effects [35]. For this reason, it is important to search for alternatives that have a restorative effect and at the same time do not cause bone loss [36]. In addition, the often long-lasting severe pain in the musculoskeletal system caused by persistent chronic inflammatory processes underscores the need for prophylactic integral treatment options to maintain the quality of life for many OP patients [37].

The multitude of different factors correlating with the development of OP may explain the large treatment gap of this disease [15,22]. These range from risk factors for primary OP, such as reproductive hormone deficiency, especially estrogen deficiency in menopausal women; mineral or vitamin deficiencies such as calcium, vitamin D, or vitamin K2; and lack of physical activity [33], to numerous chronic primary diseases such as autoimmune diseases, allergies, and various cancers, leading to secondary OP [15,33]. In addition, many medications to treat these underlying chronic inflammatory diseases, including anticancer drugs, can disrupt the mechanism of bone remodeling and cause secondary OP [38]. Importantly, in many cases, all of these risk factors have been found to correlate with dysbiosis [39], which is a high-risk factor for OP itself [40]. 

Microstructurally, OP is characterized by reduced extracellular matrix components, including decreased levels of type I collagen produced by osteoblasts. In healthy bone, there is a delicate balance between bone resorption by osteoclasts and bone formation by osteoblasts, referred to as bone remodeling, orchestrated mainly by mechanosensible osteocytes [15]. Dysregulation of bone remodeling is based on over-activation of inflammatory pathways, including NF-κB and receptor activator of NF-κB (RANK)/RANKL [41], associated with increased osteoclastogenesis and decreased activity of anabolic pathways, such as Wingless (Wnt) [42]. In this regard, several lifestyle factors such as smoking, hypercaloric diet, and physical inactivity are widely recognized to promote NF-κB activation [43]. Therefore, an optimal patient-tailored treatment strategy must identify and reduce pro-inflammatory risk factors as part of an integral OP treatment regimen that bidirectionally addresses the bone remodeling process by modulating osteoclastogenesis and promoting intrinsic bone regeneration by actively stimulating the expression of osteogenic transcription factors such as Runx2 [44]. Although there is an increasing awareness of the influence of chronic inflammatory processes that need to be addressed by multifunctional molecules, this essential component of active stimulation of osteogenesis is currently often still missing in the standard pharmacological management of OP, which essentially focuses on the administration of bisphosphonates, monospecific antibodies against RANKL, or other mono-targeted antibodies such as Sclerostin inhibitors [36].

The clinical fact that dysbiosis is the common denominator of the aforementioned risk factors for primary and secondary OP also remains undiagnosed and untreated in most cases. However, imbalances in the GM are increasingly recognized to have pro-inflammatory and detrimental effects on the entire organism, including bone tissue along the gut–bone axis [9].

## 3. Gut Microbiota and Resveratrol

First GM was discovered at the end of the 19th century [45], but their importance to the human organism in terms of their symbiotic function in promoting health, as well as their significance in the continually increasing number of chronic diseases, is only beginning to be recognized in the interdisciplinary focus [2]. 

GM colonization is primarily initiated during birth through the natural birth canal; however, it is hypothesized by current research groups that the GM is partially formed or influenced prenatally [46]. After birth, individual GM is further modified by factors such as breastfeeding and subsequently by various environmental factors, including geography, endogenous factors such as aging as well as by numerous lifestyle factors including medications, circadian rhythm, dietary habits, physical activity, emotional and mental resilience and many more [47]. All of these factors affect the GM by promoting the growth and diversity of either beneficial GM or harmful species. It is therefore an integral principle for long-term health to be aware of these factors and to choose a lifestyle accordingly.

Generally, a high diversity of beneficial bacteria, including *Bacteroidetes*, *Firmicutes*, *Actinobacteria*, *Proteobacteria*, *Fusobacteria,* and *Verrucomicrobia* has been found as part of a healthy GM [1]. In this regard, many variations are suggested and there is no specific standardized definition for a healthy GM composition [48]. However, it is widely understood that in healthy individuals, the large intestine harbors most GM with approximately 10^12^ microbial cells/gram, whereas the small intestine has been found with only approximately 10^3^–10^7^ cells/gram and less diversity, highlighting GM’s optimal adapted symbiotic functioning to support the different intestinal sections [49,50]. In this regard, GM in the small intestine has been demonstrated to modulate digestive and absorptive processes from dietary lipids [50], and GM in the large intestine has integral functioning for several systemic processes such as digestion, blood pressure, and glucose metabolism over multiple described gut–organ axes. In these interactions, GM metabolites are important actors in the reciprocal communication between the gut and other organs [39]. In fact, a healthy GM composition is characterized by a sufficient level of beneficial bacterial metabolites in accordance with an anti-inflammatory environment in the gut [1,4], which is discussed in more detail in the next subchapter. 

### 3.1. Cross-Talk between Microbiota and Organs

Although many correlations between the GM and organs are still largely undiscovered, a growing body of evidence suggests various mechanisms of reciprocal influence. First of all, this becomes evident from the fact that the GM encodes a large number of genes that are absent in the human genome, including genes for the synthesis of vitamins including soluble B vitamins and vitamin K2, emphasizing the beneficial role of GM in providing a balanced micronutrient status for the organism [51]. Furthermore, the GM plays an important role in modulating the maturation of the mucosal immune system, supporting the significance of the GM as approximately 70–80% of the immune system resides in the gut [7,8]. Interactions between vitamin D and GM are an important bidirectional axis in terms of anti-inflammatory conditions, where vitamin D has been shown as an essential factor in maintaining a physiological GM balance [5]. Additional interesting aspects have been shown in terms of reciprocal interactions between the GM and hormones, including interactions with parathyroid hormone [52] and estrogen metabolism [53], which will be addressed in more detail within Section 3.2 and Section 3.1.2. and in the context of bone health and the development of OP.

GM is recognized to produce several bioactive substances that benefit the entire organism by modulating inflammatory processes [9,54]. Currently, the best-known beneficial GM metabolites are SCFA, such as acetate, propionate, and butyrate [55], which have been implicated in many beneficial aspects, including regulation of energy homeostasis [56], regulation of pH, and maintaining the intestinal barrier [57]. SCFA are produced mainly by GM of the large intestine through fermentation of indigestible dietary fiber, evidenced by positive correlations between elevated levels of circulating SCFA and high-fiber diets in clinical trials [58]. The functional link between SCFA and organs has been demonstrated by anti-inflammatory, anti-oxidant, and beneficial anabolic effects, which stimulate tissue regeneration [59], and catabolic anti-cancer effects [60].

Overall, many positive effects have been shown in association with appropriate levels of GM-derived SCFA, including reduced blood pressure [61], cognitive function [62] as well as regulation of bone mass [63]. In addition, there is a decisive interaction between GM and immune cells, as clinically evidenced by protection against graft-versus-host disease in conjunction with appropriate levels of butyrate and propionate [64]. In particular, SCFA have been shown to stimulate the expression of interleukins (ILs), including IL-22, by CD4^+^ T-cells and innate lymphoid cells via the mTOR and Stat3 signaling pathways [65]. Of great clinical importance is the multimodal function of GM metabolites, particularly SCFA, in an epigenetic context, as discussed in the next section.

#### 3.1.1. Epigenetic Modulation

Epigenetics refers to acquired lifelong reversible modifications in gene expression that occur independently of changes in the DNA sequence influenced by individual lifestyle habits including physical activity, nutritional patterns, mental and emotional resilience, and many other factors [15]. In addition, recent evidence suggests that epigenetics can be inherited across generations and that diet during pregnancy can have an impact, for example, polyphenol intake [66]. This is also supported by the fact that chronic inflammation in the mother can influence the genetic diversity and taxonomy of GM in the offspring [67].

In terms of harmful or favorable stimulants, the activation of mechanisms for DNA reparation and intrinsic tissue regeneration processes are stimulated by different epigenetic regulatory mechanisms including DNA methylation and hydroxymethylation, histone modification, and chromatin remodeling [68]. GM metabolites such as SCFA have been found to modulate histones through acetylation or deacetylation and regulation of non-coding RNA such as microRNA (miRNA) [69] associated with the modulation of major signaling pathways such as NF-κB and Wnt both locally in the gut cells as well as systemically in multiple organs [9,54]. Additionally, SCFA have been demonstrated to modulate the NF-κB pathway in macrophages associated with the regulation of pro-inflammatory cytokines [57]. Furthermore, modulation of pro-inflammatory T-cells by promoting Treg cells and inhibiting T-helper cells 17 (Th17) by SCFA has been found [3]. Interestingly, and of high clinical relevance, GM is also known to indirectly influence epigenetics by metabolizing phytochemicals such as epigenetically active polyphenols, thereby further contributing to an anti-inflammatory microenvironment both locally and systemically via the bloodstream [30]. This is discussed further in Section 3.5.3. as an integral preventive and adjunctive treatment regimen in terms of OP. 

#### 3.1.2. Gut–Bone Axis

The gut–bone axis refers to the multi-facetted interplay between the GM and bone cells, which have been shown to communicate directly with each other via a variety of GM-derived metabolites such as SCFA that can epigenetically modulate crucial pathways in bone remodeling, including Wnt and NF-κB, as described in the previous subsection. With specific regard to bone cells, SCFA have been demonstrated to promote bone formation by stimulating osteogenic transcription factors such as Runx2 [70]. Especially, acetate and propionate have been found to up-regulate early osteogenic markers, such as alkaline phosphatase (ALP) [55]. In this regard, acetate has been shown to up-regulate ALP mRNA, supporting its epigenetic functioning within bone tissue [55]. Concurrently, butyrate has also been demonstrated to regulate pro-inflammatory markers associated with attenuated osteoclast activity and improved bone regeneration processes [59]. In addition, butyrate has been shown to stimulate osteogenesis by increasing intestinal and bone marrow-derived Treg-cells, which have been shown to up-regulate Wnt10b expression in CD8^+^ T-cells [8]. In general, the GM has been shown to be a critical factor for a physiological Treg/Th17 ratio in terms of maintaining balanced bone remodeling [3]. 

Interestingly, SCFA have further been found to influence bone formation by interacting with hormones such as estrogen and parathyroid hormone mentioned in the previous chapter. In this regard, specific gut bacteria, known as estrobolome, have been found to be able to produce the enzyme β-glucuronidase, enabling them to reactivate estrogen for recirculation through blood stream, thereby modulating systemic levels of active estrogen [6]. Concurrently, the physiological levels of estrogen have been shown as a key player in promoting a beneficial GM diversity supporting a gut–estrogen axis [71]. Further evidence of positive GM-hormone cross-talk is the interaction with parathyroid hormone which requires SFCA for osteogenesis [52]. Remarkably, GM has also been shown to play an important role in modulating bone remodeling via modulation of tryptophan and attenuation of peripheral serotonin levels, leading to stimulation of osteogenic transcription factors such as FoxO1 [72,73]. Preclinically, GM has further been demonstrated to stimulate the expression of Insulin-like growth factor (IGF)-1, thereby promoting osteogenesis in bone tissue [74]. 

GM further influences bone remodeling through interaction with key factors required for bone formation, including vitamin D and calcium resorption and the production of vitamin K2 [23]. This is of great clinical importance as many OP treatment regimens include supplementation of these factors. However, some clinical findings are contradictory as no additional beneficial effects of calcium supplementation have been shown [75], which could indicate malabsorption due to an inflamed intestinal barrier characterized by loss of tight junctions [76]. Supportive evidence for the impact of GM on optimal calcium resorption comes from findings demonstrating that probiotics with *Lactobacilli* improved calcium absorption, which was associated with a decrease in pro-inflammatory cytokines [76]. Therefore, the maintenance of an anti-inflammatory gut microenvironment characterized by an intact intestinal barrier is recognized as an essential factor for bone health. This environment supports bone cells by preventing pro-inflammatory components from food and metabolic products of harmful bacteria such as lipopolysaccharide (LPS) and TMAO from entering the bloodstream [3].

Altogether, there are a variety of mechanisms by which GM communicates with bone cells and consequently, the dysregulation of the gut–bone axis has been implicated in several pathological conditions, including OP, and a large number of risk factors have been identified, which will be reviewed in the next subchapter.

### 3.2. Risk Factors for Developing Dysbiosis

Dysbiosis refers to the disruption of the natural balance of GM, resulting in pathogenic GM overgrowing and beneficial GM outcompeting. In this regard, dysbiotic GM containing *Escherichia coli*, *Klebsiella*, and *Shigella* have been reported with higher abundance [77] while beneficial GM including *Akkermansia muciniphila* and *Faecalibacterium prausnitzii* are reduced [78]. 

**Figure 1:**
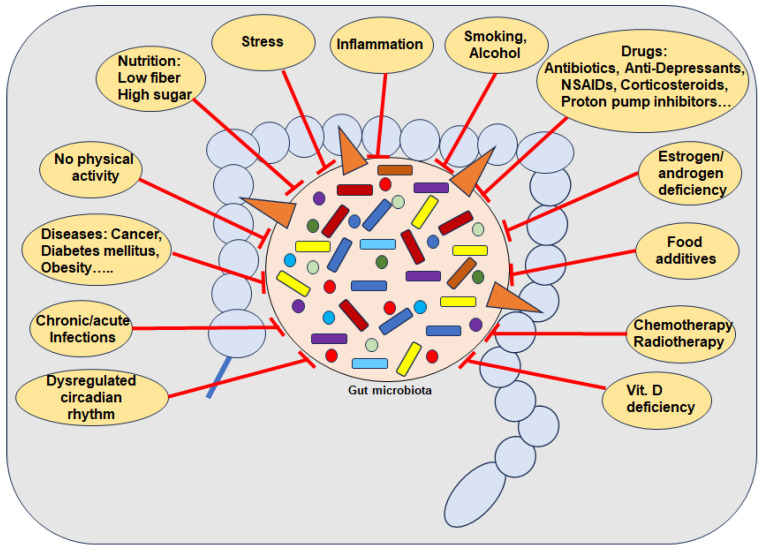
Several causes for the depletion of beneficial gut bacteria. Abbreviations: NSAIDs—Nonsteroidal anti-inflammatory drugs, Vit—Vitamin.

Numerous external pro-inflammatory lifestyle factors are known as risk factors for the development of dysbiosis, including a diet low in fiber and high percentage of simple carbohydrates [54]; food additives [79]; disrupted circadian rhythm [80]; smoking [81]; alcohol [82]; lack of physical activity or long-term intense training [11], vitamin D deficiency [5] and even drugs such as antibiotics and many non-antibiotic drugs including PPIs [83], NSAIDs [16] and chemo- and radiotherapy [19] (Figure 1). Importantly, dysbiosis has further been found by several aging processes associated with estrogen deficiency [84]. Indeed, in germ-free mice with reproductive hormone deficiency, it was clearly demonstrated that no bone loss occurred [53]. Of note, besides postmenopausal women, breast cancer patients undergoing hormone therapy such as aromatase inhibitors as well as localized or metastatic prostate cancer patients treated with androgen deprivation therapy and/or new androgen receptor pathway inhibitors, are also affected [29,85]. 

Regarding the time period between measurable changes in GM and the onset of risk factors, there is much research indicating a very short time period associated with pro-inflammatory dietary habits and changes in GM composition [10]. In this context, a correlation between a 2-day high-simple carbohydrate diet and decreased protective SCFA with increased gut permeability has been demonstrated, heightening susceptibility to colitis in mice by inducing inflammation and impairing tissue repair mechanisms [54]. Interestingly, it has been shown that dysbiosis is negatively correlated with total calorie intake, but rather the percentage of the various macronutrients is decisive, whereby in particular a high proportion of simple carbohydrates showed negative effects on GM, which is known for many ultra-processed foods [54,79] (Figure 1). Additional supportive evidence has been provided by a randomized crossover study demonstrating that foods with a high percentage of simple carbohydrates lead to severe GM changes after 4 days compared to a GM proactive Mediterranean diet [86]. Various artificial sweeteners have also been shown to lead to an imbalance in GM within 5 days [87], associated with induced glucose intolerance [88], which supports the hypothesis that it is not the calorie intake but the individual ingredients that are decisive. Further clinical evidence shows that patterns of duration of food intake affect the composition of the GM, as intermittent fasting for about 16 h increases the diversity of the GM, including the *Lachnospiraceae* that produce butyric acid [89]. This supports clinical findings that poor glycemic control, reflected by high levels of HbA1c percentages, has been associated with dysbiosis [90] (Figure 1). Overall, the data suggest that the GM can change rapidly through diet, even within a single day with certain dietary changes. However, factors such as current GM diversity, underlying diseases, and the wide variation in food quality must be considered as additional influencing variables. [10]. It is therefore logical to assume that dysbiosis is linked to a multitude of chronic inflammatory diseases including OP, clinically evidenced by the observed fact that GM composition positively correlates with both health and disease markers [91]. 

#### Drug-Microbiota-Interaction

The negative impact of certain medications on the GM is increasingly recognized as a significant concern in clinical practice and it has been concluded that the individual GM is a reflection of the combinations of medication taken by the individual [16]. 

Antibiotics, widely used to treat bacterial infections, are known to negatively affect the GM. Specifically, it has been clinically demonstrated in healthy individuals that although GM diversity mostly recovers within 6 weeks, at least nine common GM species that were present before treatment remain absent even 25 weeks after completing a 4-day course of antibiotic therapy [17]. Notably, it has been reported that both a severe alteration of the GM with a major loss of diversity as well as a decrease in individual beneficial bacterial species that are synergistically essential for the production of anti-inflammatory SCFA are of particular importance for the prevention and treatment of chronic inflammatory diseases [92], as evidenced by increased susceptibility to pathogens in the absence of single specific beneficial GM [54]. Moreover, antibiotics may alter estrogen metabolism linked with a reduction in GM diversity, supporting bi-directional interactions and emphasizing the importance of an integrative paradigm to identify the primary root factors of OP [93]. However, recovery compounds such as pre- and probiotics are often not yet included in the standard follow-up treatment. 

Similarly, a variety of non-antibiotic drugs have been found to be associated with reductions in microbial diversity and shifts in taxonomic abundance including long-term use of PPIs for conditions such as gastroesophageal reflux disease and peptic ulcer disease [18]. Likewise, NSAIDs, which are among the most frequently self-prescribed medications have further been shown a significant negative impact on GM composition in various independent studies [83]. Notably, the bacterial composition of the gut varied with the type of NSAID ingested [16]. In addition, corticosteroids, including long-term prednisone, have also been shown to alter GM composition by promoting fungal overgrowth [94]. Antidepressants, notably selective serotonin reuptake inhibitors, also significantly reduce genetic GM diversity [95]. This highlights the paradox of most current standard treatments, as numerous neurological diseases have been shown to correlate with dysbiosis even before medication [96], increasing the risk of numerous metabolic secondary diseases. Moreover, many anti-cancer drugs such as anthracyclines, selective estrogen receptor modulators (SERMs) and Poly(adenosine diphosphate [ADP]-ribose) polymerase (PARP) inhibitors used in breast cancer have been demonstrated to reduce GM diversity [97]. This is also of great importance for patients with breast cancer with underlying dysbiosis, which may be further exacerbated by hormone therapies including aromatase inhibitors [98]. Also of significance, the use of laxants such as during coloscopies has been found to correlate with reduced GM diversity and it has been suggested to prescribe probiotics after treatment [99]. 

### 3.3. Significance of Dysbiosis in Chronic Inflammatory Diseases

**Figure 2:**
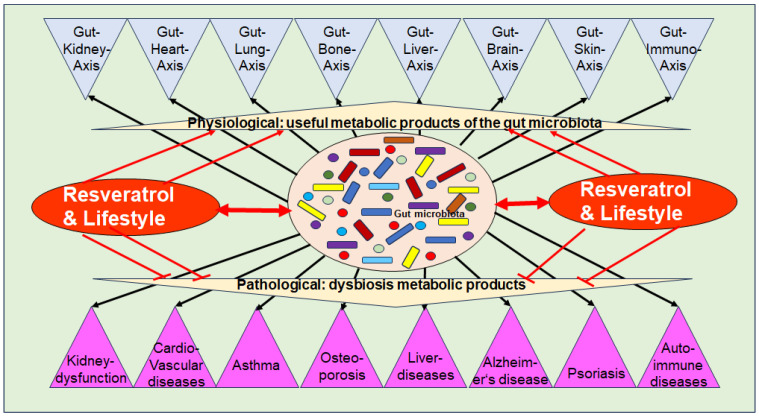
Functional axis between the intestinal microbiota, its metabolic products, and numerous body organs and chronic diseases.

Dysbiosis has been implicated in various chronic inflammatory diseases, including kidney dysfunction, cardiovascular diseases, asthma, liver diseases, auto-immune diseases, numerous types of cancer, metabolic syndrome as well as musculoskeletal diseases such as OP [91] (Figure 2). As outlined in Section 3.1, intact organ functioning is dependent on a high diversity of beneficial GM-producing multiple bioactive metabolites, which modulate crucial inflammatory pathways, along with the modulation and maintenance of an intact immune defense. In addition, catabolic anti-cancer effects have been identified [60]. These regulatory mechanisms between GM and organ tissues are defined as specific gut–organ axes, such as the gut–brain axis [100], the gut–liver axis [101], the gut–skin axis [102], and the gut–bone axis [103] (Figure 2).

Evidence suggests that dysbiosis is involved in the induction and exacerbation of chronic inflammation, which is characterized by the persistent expression of pro-inflammatory cytokines via constitutive activation of inflammatory pathways such as NF-κB, which is a crucial hallmark in the development of many chronic diseases, including OP [9]. Notably, inflammation-induced micro-damage to intestinal epithelial cells, leading to disruption of the intestinal barrier, commonly referred to as “leaky gut” which in turn contributes to systemic inflammation [9]. In this regard, overgrowth of pro-inflammatory GM has been demonstrated to produce several pro-inflammatory metabolites including the endotoxins TMAO and LPS, which are activators for pro-inflammatory signaling such as NF-κB [9] (Figure 2). Furthermore, activation of other pathways such as histamine H1 receptor signaling, has been found to be induced by pro-inflammatory GM-derived products, such as LPS correlating with cancer growth [104].

It should be emphasized, that dysbiosis is associated with several types of cancer outside the intestinal tract such as brain tumors [105] and breast cancer [6]. In the context of breast cancer, dysbiosis is discussed as a central factor for chronic inflammation and as an important factor influencing hormone metabolism, in which the aforementioned estrobolome is involved, especially in hormone-sensitive breast cancer through the reactivation of estrogen, which increases its serum level systemically [6] which has further been confirmed for prostate cancer associated with GM species capable of converting androgen precursors into active androgens, thereby contributing to endocrine resistance by providing an alternative source of androgens [106].

Considering the fact that the GM also metabolizes drugs, including several anti-cancer drugs, the maintenance and active promotion of a beneficial GM is of great clinical interest. This hypothesis is clinically evidenced by findings that dysbiosis itself is a critical factor in the clinical outcome of various anti-cancer therapies, such as anti-CD19 chimeric antigen receptor (CAR) T-cell therapy [107]. The response to CD19 CAR T-cell therapy has been shown to correlate with altered GM composition including *Ruminococcus*, *Bacteroides*, and *Faecalibacterium* [107]. A link to a poorer treatment outcome has been demonstrated in patients receiving antibiotics, particularly broad-spectrum antibiotics, prior to CAR T-cell infusion therapy [107]. Supporting the essential role of GM in terms of therapy outcome, immune checkpoint inhibitor therapies have been demonstrated to be more effective with the parallel targeting of the GM [108].

According to current knowledge, OP is one of the most significant long-term side effects of many chronic inflammatory diseases, and chronic drug treatments and continues to pose a particular challenge to women’s health due to the post-menopausal state characterized by estrogen deficiency. Interestingly, GM has been shown to play a crucial role in promoting an imbalance in bone remodeling through a disturbed gut–bone axis (Figure 2), which is discussed in the following chapter.

### 3.4. Dysbiosis-Induced Osteoporosis

Emerging evidence suggests a link between gut dysbiosis and OP development via the gut–bone axis. Dysbiosis is associated with impaired intestinal barrier function, leading to increased circulation of endotoxins and other pro-inflammatory mediators, which in turn affect physiological bone remodeling by stimulating osteoclastogenesis and inhibiting osteogenesis. Preclinical evidence supports this hypothesis, such as results showing that transferred gut dysbiosis from senile OP in vivo models induces OP in young animals [109]. In parallel, clinical studies confirm commonality in OP patients and that its targeted therapeutic treatment correlates with positive effects on bone metabolism [23]. Therefore, the individual GM is suggested a biomarker for bone remodeling. Interestingly, estrogen deficiency appears to be one of the biggest risk factors for widespread dysbiosis in postmenopausal women [110]. It is postulated that the condition of estrogen deficiency and dysbiosis is involved in many pathological processes, especially in dysregulations of immune cells, including an increased number and activity of mast cells, which is confirmed by the evidence of overactivation of mast cells in a state of estrogen deficiency [71,111]. This assumption is supported by evidence from other research groups that have demonstrated an increase in mast cells in the bone marrow of postmenopausal women in clinical studies, which is consistent with reproducible preclinical in vivo study models [41]. In this context, histamine is considered one of the central molecules released by degranulating mast cells, which has also been shown to be an important signaling molecule of the GM [112]. Indeed, *Citrobacter* and *Morganella*, both capable of producing histamine, have been associated with low bone mass [113]. The hypothesis that histamine is a major contributor to bone loss is further supported by clinical evidence that individuals undergoing drug treatment with anti-histamines have been shown in association with increased bone density [114]. However, this clinical trial neglected the consideration of postulated underlying gut dysbiosis.

Clinically, many OP patients also show a correlation with alterations in GM-derived metabolites, including decreased beneficial SCFA [71] and increased levels of harmful GM-derived metabolites such as pro-inflammatory TMAO linked with less diversity of beneficial GM [84]. The positive correlation between increased levels of TMAO and increased risk of hip fractures in postmenopausal women further supports the hypothesis of the harmful effects of TMAO on bone health [115]. Activation of NF-κB in MSC is well recognized to suppress osteogenic transcription factors such as Runx2 via Sirt1 inactivation [24] and preclinical evidence demonstrated that TMAO promotes osteoclastogenesis via induction of ROS-dependent NF-κB signaling pathway [116] and impairs osteogenesis by stimulating mesenchymal stem cells (MSC) to differentiate into adipocytes instead of osteoblasts [117]. Likewise, indoxyl sulfate has been demonstrated as another harmful pro-inflammatory GM metabolite in terms of decreasing the expression of Runx2 via the aryl hydrocarbon receptor/p38 mitogen-activated protein kinase (MAPK) signaling pathway [118]. Moreover, bile acids have been implicated by acting on osteoblasts and osteoclasts via bile acid-activated receptors associated with increased bone resorption processes, demonstrated in dysbiotic conditions [119]. Furthermore, altered amino acid metabolism, including tyrosine and tryptophan, as well as valine, leucine, and isoleucine degradation, correlates with microbiota biomarkers and OP [120]. There is also growing evidence that GM interacts with mRNAs and thereby modulates bone remodeling epigenetically [121], which is of great importance as dysregulation of mRNAs has been found in many cases of OP [15]. 

#### Identity of Microbiome Related to OP

**Figure 3:**
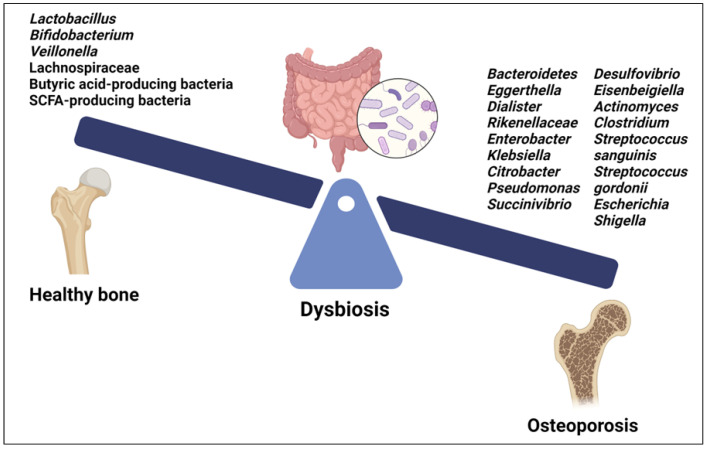
Microbial signatures associated with osteoporosis. Created with BioRender.org https://app.biorender.com/ (accessed on 1 May 2024).

Recent studies have begun to elucidate the specific microbial signatures associated with OP. Both gut and oral microbiota are involved in modulating bone health through intricate crosstalk with the host immune system and regulation of inflammatory pathways [9,122] (Figure 3).

Among the GM, certain species of *Lactobacillus* and *Bifidobacterium*, as well as their metabolites, have beneficial effects on bone health (Figure 3). They improve BMD and reduce the risk of OP by promoting intestinal calcium absorption and modulating the activity of osteoclasts and osteoblasts. Moreover, some *Lactobacillus* and *Bifidobacterium* species have been found to modulate immune responses [9]. The GM of OP patients showed decreased levels of *Lactobacillus*, *Bifidobacterium*, and butyric acid-producing bacteria along with increased levels of pathogenic bacteria such as *Clostridium* and some species of *Streptococcus* and *Actinomyces*, including *Streptococcus sanguinis, Streptococcus gordonii* and *Actinomyces odontolyticus* [123] (Figure 3). Furthermore, a positive correlation was found between an abundance of *Bacteroidetes*, *Eggerthella*, *Dialister*, *Rikenellaceae*, *Enterobacter*, *Klebsiella*, *Citrobacter*, *Pseudomonas*, *Succinivibrio*, *Desulfovibrio*, and *Eisenbeigiella* and decreased BMD, bone loss, and OP risk [113,123] (Figure 3). Changes in certain gut bacteria among individuals with OP were associated with vitamin D metabolism; higher levels of *Escherichia* and *Shigella* were observed in individuals with osteopenia and postmenopausal OP, suggesting a correlation with vitamin D deficiency, which contributes to bone loss [124,125]. Moreover, *Eggerthella* species have been implicated in potentially diminishing the quantity of vitamin D receptors, contributing to bone loss linked with OP [124,125] (Figure 3).

Several in vivo studies have shown a benefit for certain probiotics as a treatment for OP. For example, *Lactobacillus rhamnosus* (*L. rhamnosus*) decreases the percentage of osteoclastogenic CD4^+^Rorγt^+^ -Th17) and enhances anti-osteoclastogenic CD4^+^Foxp3^+^ Tregs and CD8^+^Foxp3^+^Tregs, thereby attenuating bone loss and enhancing bone microarchitecture in ovariectomized mice [126] (Table 1). A recent study showed that *L. rhamnosus* GG stimulates Th17/Treg balance and reduces osteoclastogenic inflammatory cytokines in both the gut and bone, attenuating bone loss and helping ameliorate OP [127] (Table 1). Similarly, *Lactobacillus acidophilus* (*L. acidophilus*) modulates the balance between Treg and Th17 cells, inhibiting osteoclastogenic Th17 cells and promoting anti-osteoclastogenic Treg cells [128] (Table 1). In addition, *L. rhamnosus* and *L. acidophilus* administration led to a reduction in osteoclastogenic cytokines, such as RANKL, IL-6, IL-17, and TNF-α, and to an increase in anti-osteoclastogenic factors such as IL-4, IL-10, and IFN-γ in ovariectomy-induced postmenopausal mice models [126,127,128,129]. Interestingly, Chen et al. demonstrated that *L. rhamnosus* and *L. acidophilus* supernatant stimulate proliferation, differentiation, and maturation of pre-osteogenic MC3T3-E1 mouse cells and reduced proliferation of osteoclast progenitor RAW 264.7 cells, at least in part through the production of butyric acid [130]. Another recent study showed that *L. acidophilus* inhibits osteoclast formation and bone resorption activity by producing butyrate in ovariectomized mouse models [131]. 

*Lactobacillus reuteri* (*L. reuteri*) seems to be especially promising as a treatment for OP. It improved postmenopausal OP by suppressing osteoclastogenesis through the reduction of osteoclast-inducing signals from CD4^+^ T-cells [132] (Table 1). Interestingly, Collins et al. reported that the administration of *L. reuteri* increased bone density in male wild-type mice but not in Rag knockout mice, which lack mature T- and B-lymphocytes, suggesting a critical role of lymphocytes in mediating these effects. Moreover, T-cells from mesenteric lymph nodes treated with *L. reuteri* supernatants secrete cytokines, such as IL-10, that enhance osterix expression, thereby promoting MC3T3-E1 osteoblast differentiation [133] (Table 1). A recent study demonstrated that simultaneous supplementation with *L. reuteri* and calcium fluoride nanoparticles promotes an increase in osteoblasts and osteocytes, accompanied by a rise in serum estrogen levels and a decrease in serum calcium and ALP levels [134] (Table 1). 

*Lactobacillus casei (L. casei*) is one of the most extensively researched probiotics, and several preclinical studies have demonstrated that treatment with *L. casei* can alleviate bone loss. *L. acidophilus* and *L. casei* significantly improved calcium and ALP levels while decreasing phosphorus content, increasing vitamin D, and enhancing BMD, bone marrow concentration, and bone area in ovariectomized rats [135] (Table 1). Guo et al. found that *L. casei* fermented milk promotes fracture healing and increased callus formation in osteoporotic mice following antibiotic-induced dysbiosis by inhibiting the RAS/RANKL/RANK pathway [136] (Table 1). Likewise, fermented milk containing *L. casei* 393 enhanced the proliferation of osteoblastic MC3T3-E1 cells and boosted bone weight, BMD, and bone-breaking force in ovariectomized rats [137]. Additionally, the extract of *L. casei* effectively inhibited the expression of osteoclast-specific genes, such as cathepsin K, TRAP, calcitonin receptor, and integrin β3, and down-regulated key transcription factors such as c-Fos, NFATc1, and NF-κB, leading to the suppression of osteoclast differentiation and bone resorption in ovariectomized rats [138] (Table 1). 

*Lactobacillus plantarum* (*L. plantarum*) also shows potential in treating OP. *L. plantarum* treatment increased gut microbial diversity and beneficial bacteria abundance, thereby improving bone density and microstructure and inhibiting osteoclast differentiation [139] (Table 1). Interestingly, the *L. plantarum* strains AR237 and AR495 differ in their effectiveness in treating OP. AR495 strain was more effective than AR237 in reducing osteoporotic fractures and inhibiting bone resorption by modulating the RANKL/RANK/OPG and TLR4/Myd88/NF-κB pathways. Moreover, AR495 increased the abundance of SCFA-producing bacteria in the feces and intestines, which may be, at least in part, responsible for its anti-osteoporotic effects in ovariectomized mice [140] (Table 1). Other Lactobacillus strains, such as *Lactobacillus paracasei* and *Lactobacillus brevis*, hindered bone loss in ovariectomized mice by reducing the production of pro-inflammatory markers and inhibiting osteoclastogenesis [141,142] (Table 1).

Compelling evidence indicates that in addition to lactic bacteria, certain strains of *Bifidobacterium* may exert beneficial effects on bone health by modulating the GM and influencing systemic immune responses. These probiotic bacteria have been shown to enhance calcium absorption, reduce inflammation, and regulate bone metabolism [9,143]. For example, *Bifidobacterium* lactis BL-99 prevented OP in an experimental model of dextran sodium sulfate-induced ulcerative colitis [144] (Table 1). 

Several preclinical studies have explored the effect of *Bifidobacterium longum* (*B. longum*) on OP. By up-regulating Sparc and BMP-2 genes, *B. longum* increased BMD and decreased bone resorption in rats with bone loss due to ovariectomy [145] (Table 1). Interestingly, *B. longum* also exhibited immunomodulatory potential in an ovariectomy-induced osteoporotic mouse model by enhancing the differentiation of regulatory B-cells and consequently modulating the balance of Treg–Th17 cells. Furthermore, *B. longum* suppressed the functional activity of RANKL-induced osteoclastogenesis in both mouse bone marrow cells and human PBMCs [146] (Table 1). In the same line, the co-administration of *B. longum* NK49 and *L. plantarum* NK3 alleviated OP by down-regulating NF-κB-associated TNF-α expression via modulation of GM in ovariectomized mice [147] (Table 1). In a further study, Gholami et al. demonstrated that among nine effective probiotics, the combination containing *L. acidophilus*, *L. reuteri*, and *B. longum* showed the most significant ameliorative effect on bone density and mineral content in ovariectomized rats [148] (Table 1).

Other bacteria have also demonstrated a strong potential as a probiotic treatment for bone health. In a mouse model of postmenopausal OP, *Bacillus clausii* improved bone health by shifting the balance of Treg-Th17 cells, reducing pro-inflammatory cytokines, and increasing anti-inflammatory factors [149] (Table 1). Additionally, Yuan et al. demonstrated that *Bacteroides vulgatus* oral treatment in ovariectomized mice improved microbiota dysbiosis, reduced inflammation by inhibiting the LPS/TLR-4/NF-κB pathway, and ameliorated lumbar bone loss [150] (Table 1). Remarkably, *Bacteroides* species also facilitate the absorption of vitamin K2, which serves as an essential cofactor for bone formation and overall bone health [151]. 

Certain species of *Prevotella* have also been associated with bone homeostasis, inflammatory response, and OP [152] (Table 1). For instance, in mice with ovariectomy-mediated OP, *Prevotella histicola* perfusion mitigated bone loss by repressing osteoclastogenesis, promoting osteogenesis, lowering pro-inflammatory cytokines, such as IL-1β and TNF-α, and improving the composition, abundance, and diversity of GM [152]. Additionally, in contrast with other *Clostridium* species, *Clostridium butyricum* decreased GM-induced bone resorption by enhancing autophagy mechanisms in osteoblasts [153]. Finally, *Akkermansia muciniphila* was directly associated with the process of bone formation and OP attenuation [154], supporting the clinical observation that the abundance of *Akkermansia muciniphila* is reduced in patients with osteopenia compared to the control group [155].

The oral microbiota comprises a diverse community of bacteria, fungi, and viruses residing in the oral cavity. While primarily associated with oral health and disease, emerging evidence suggests a potential link between oral microbiota dysbiosis and systemic conditions, including OP. Several pathogens implicated in periodontal disease, such as *Porphyromonas gingivalis*, *Aggregatibacter actinomycetemcomitans*, and *Fusobacterium nucleatum* have been associated with systemic inflammation and bone loss, possibly mediated through the release of pro-inflammatory cytokines and activation of osteoclasts [122]. In addition, metabolites produced by oral microorganisms, such as SCFAs, organic acids, and enzymes, may enter the systemic circulation and influence distant organ systems, including bone tissue [122].

**Table 1:** Preclinical effects and mechanisms of probiotics on bone remodeling and health in OP.

GM Genus	GM Species	Experimental Model	Mechanisms of Action	Effects	Reference
*Lactobacillus*	*L. rhamnosus*	OVX mice	↓ CD4^+^Rorγt^+^Th17 cells ↑ CD4^+^Foxp3^+^Tregs ↑ CD8^+^Foxp3^+^Tregs ↓ IL-6, IL-17 and TNF-α ↑ IL-4, IL-10 and IFN-γ	↓ Osteoclastogenesis ↓ Bone loss ↑ Bone microarchitecture	[126]
	*L. rhamnosus GG*	OVX rats	↓ CD4^+^IL-17A^+^Th17 cells ↑ D4^+^CD25^+^FOXP3^+^Treg cells ↓ IL-17 and TNF-α ↑ IL-10 and TGF-β	↑ Osteogenesis ↑ Bone microstructure and biomechanics ↓ Estrogen deficiency-induced OP	[127]
	*L. acidophilus*	OVX mice	↓ Th17 cells ↑ Tregs cells ↓ IL-6, IL-17, TNF-α and RANKL ↑ IL-10 and IFN-γ	↑ Bone microarchitecture ↑ BMD ↑ Bone heterogeneity	[128]
	*L. rhamnosus* and *L. acidophilus*	MC3T3-E1 and RAW 264.7 cells	↑ ALP, osteocalcin, RUNX2, NFATc1, cathepsin K, DC-STAMP, OSCAR, Wnt2, and CTNNB1 in MC3T3-E1 ↓ RANK, NFATc1, cathepsin K, DC-STAMP, OSCAR, Wnt2, and CTNNB1	↑ Proliferation, differentiation, and maturity of MC3T3-E1 cells ↓ Proliferation, differentiation, and maturity of RAW 264.7 cells	[130]
	*L. acidophilus*	OVX C57BL/6 J mice	↓ B cells ↓ Production of RANKL on B- cells ↑ Butyric acid levels	↓ Osteoclast formation ↓ Bone resorption activity ↓ Systemic bone loss	[131]
	*L. reuteri* ATCC PTA 6475	OVX mice	↓ Trap5 and RANKL ↓ CD4^+^ T-lymphocytes in bone marrow GM modification	↓ Bone loss ↓ Osteoclastogenesis	[132]
	*L. reuteri* 6475	In vivo: Male WT and Rag KO mice Ex vivo: MLN and CD3+ T-cells In vitro: MC3T3-E1 cells	↑ IL-10 and IFN-γ ↑ Osterix expression	↑ Bone density in male WT mice ↑ MC3T3-E1differentiation	[133]
	*L. reuteri* 6475 + calcium fluoride NP	OVX rats	↑ Serum estrogen levels ↓ Serum calcium levels ↓ ALP serum levels	↓ Bone loss ↑ Tibial and femoral lengths ↑ Osteoblasts and osteocytes	[134]
	*L. acidophilus* + *L. casei*	OVX rats	↑ Calcium levels ↑ ALP levels ↓ Phosphorus levels	↑ BMD ↑ BMC ↑ Bone area	[135]
	*L. casei* fermented milk	Old female Kunming mice treated with antibiotics	↓ RAS/RANKL/RANK pathway	↑ Fracture healing	[136]
	*L. casei* 393 FMP	MC3T3-E1 cells and OVX rats	↓ TRAP	↑ MC3T3-E1 cells proliferation ↑ Bone weight ↑ BMD ↑ Bone breaking force	[137]
	*L. casei* extract	RANKL-induced RAW macrophage cells and OVX rats	↓ MAPK, NF-κB, c-Fos, NFATc1 ↓ Cathepsin K, TRAP, calcitonin receptor, and integrin β3	↓ Osteoclastogenesis ↓ Bone resorption activity ↓ Bone architecture alterations	[138]
	*L. plantarum*	Glucocorticoid-induced OP rats	↑ Beneficial bacteria and metabolites ↑ GM diversity	↓ Osteoclast differentiation ↓ Bone resorption	[139]
	*L. plantarum* AR495	OVX mice	↓ RANKL/RANK/OPG ↓ TLR4/Myd88/NF-κB ↑ SCFA-producing bacteria	↓ Osteoporotic fractures ↓ Bone resorption	[140]
	*L. paracasei*	OVX mice	↓ TNFα and IL-1β ↑ OPG	↓ Bone loss ↓ Osteoclastogenesis	[141]
*Bifidobacteria*	*B. lactis* BL-99	SDS-induced ulcerative colitis mice	↓ TNF-α, IL-1β, IL-6, and IL-17 ↑ Claudin-1, MUC2, ZO-1, and Occludin Changes in GM composition	↓ Bone tissue injury severity ↑ Bone volume ↑ Trabecular number and thickness	[144]
	*B. longum*	OVX-rats	↑ Sparc and Bmp-2 genes ↓ CTX ↑ Osteocalcin	↑ BMD ↓ Bone resorption ↑ Osteogenesis ↓ Osteoclasts	[145]
	*B. longum*	In vivo: OVX-mice In vitro: mouse bone marrow cells and human PBMCs	↑ CD19^+^CD1dhiCD5^+^ Bregs ↑ CD4^+^Foxp3^+^ Tregs ↑ CD4^+^IL-10^+^ Tr1 ↓ CD4^+^Rorγt^+^IL-17^+^ Th17 ↓ IL-6, IL-17, TNF-α and RANKL ↑ IL-10 and IFN-γ	↑ Bone mass and bone strength ↑ Bone microarchitecture ↓ Osteoclastogenesis	[146]
*Bifidobacterium + Lactobacillus*	*B. longum* NK49 *+ L. plantarum* NK3	OVX mice	↓ NF-κB activation ↓ TNF-α expression GM regulation	↓ OP	[147]
	*L. acidophilus + L. reuteri + B.longum*	OVX rats	↑ Serum calcium ↑ Vitamin D ↓ ALP	↑ BMD ↑ Spine BMC	[148]
*Bacillus*	*Bacillus clausii*	OVX mice	↓ Th17 cells ↑ Tregs cells ↓ IL-6, IL-17, IFN-γ and TNF-α ↑ IL-10 and IFN-γ	↓ Bone loss ↑ Bone microarchitecture ↓ Osteoclastogenesis	[149]
*Bacteroides*	*Bacteroides vulgatus* ATCC 8482	OVX female C57/BL6 mice	↓ Microbiota dysbiosis ↓ LPS/TLR-4/NF-κB ↓ TNF-α/RANKL	↓ Bone loss and microstructure destruction	[150]
*Prevotella*	*Prevotella histicola*	OVX mice and postmenopausal women	↓ IL-1β and TNF-α ↑ GM composition, abundance and diversity	↓ Bone loss ↓ Osteoclastogenesis ↑ Osteogenesis	[152]

Abbreviations: ALP—alkaline phosphatase; BMC—bone mineral content; BMD—bone mineral density; CTX—c-terminal telopeptide; FMP—fermented product; KO—knock-out; NP—nanoparticles; OVX—ovariectomized; TRAP—Tartrate resistant acid phosphatase; WT—wild-type. The up arrow (↑) indicates activation/increase/high regulation and the down arrow (↓) indicates decrease/decrease/regulation/suppression.

Clinical evidence supports the probiotic treatment of dysbiosis in postmenopausal women with beneficial GM, in particular, *Lactobacilli* [156] and Bifidobacteria [23], which are associated with improved bone markers (Table 2). Indeed, a double-blind, placebo-controlled clinical trial found that oral supplementation with *L. reuteri* 6475 significantly decreased the loss of total volumetric BMD compared to the placebo among women aged 75–80 years with low BMD [157] (Table 2). Furthermore, findings from a randomized, double-blind, placebo-controlled, multicenter trial revealed that postmenopausal women receiving a 12-month probiotic intervention containing three *Lactobacillus strains* (*L. paracasei* DSM 13434, *L. plantarum* DSM 15312, and *L. plantarum* DSM 15313) experienced a notable decrease in lumbar spine BMD loss compared to those in the placebo group [20]. Interestingly, a randomized pilot clinical trial demonstrated that *Bifidobacterium* animalis subsp. lactis Probio-M8, a novel probiotic isolated from the breast milk of a healthy woman, administered together with conventional drugs, improved bone metabolism in postmenopausal OP patients by increasing GM interactions, particularly butyrate-producing bacteria [23] (Table 2). In healthy postmenopausal women, *Bacillus subtilis* C-3102 significantly increased total hip BMD through the modulation of GM and suppressed bone resorption markers compared to the placebo group [158] (Table 2). Consequently, there was an increase in the relative abundance of *Bifidobacterium* and a decrease in *Fusobacterium*, which led to lower levels of inflammatory cytokines and reduced osteoclast activity [158]. Additional supplementation with calcium, calcitriol, and phytoderivatives such as isoflavones in addition to probiotic supplementation clinically showed an improvement in bone condition in accordance with a modulated estrogen metabolism [159] (Table 2). Prebiotic supplementation, such as the consumption of soluble corn fiber, correlated clinically with improved calcium absorption and GM composition in pubertal women [160] (Table 2). However, some studies report no clinical effects when prebiotic supplements are administered in combination with phytotherapeutics for a short period (e.g., 2 weeks) in postmenopausal women, suggesting a longer period for clinical trials [161] (Table 2). Overall, these clinical studies suggest a positive effect of combining prebiotics and probiotics with standard OP treatment regimens, which is also supported by recent clinical studies in which adjuvant treatment with traditional Chinese medicine remedies [162] or natural phytoestrogens [163] was used (Table 2). 

In conclusion, understanding the intricate interplay between GM and their metabolites in the pathogenesis of OP holds promise for the development of novel preventive and therapeutic strategies targeting GM, especially the promotion of SCFA-producing species. Taken together, the findings from all these preclinical and clinical studies suggest that supplementation with probiotics, particularly with *Lactobacillus* and *Bifidobacterium* species, is correlated with enhanced bone strength and diminished bone loss. In the long term, however, an appropriate anti-inflammatory gut environment and physiological gut motility should be sought through appropriate modifiers such as a physiological circadian rhythm, reduction of psychosocial stress, sufficient daily exercise, and a balanced and varied diet to preserve the diversity of yet unknown GM species. The overriding principle is therefore to reduce the prevailing inflammatory cascades locally in the gut while promoting the growth and diversity of beneficial GM. Daily diet is one of the most natural and rapid influencing factors, and multifunctional phytochemicals such as polyphenols seem to be an effective strategy. The subject of the next chapter addresses a promising approach with the natural polyphenol resveratrol, offering several advantages including both the modulation of the gut microenvironment and gut dysbiosis and active stimulation of the intrinsic bone regenerative capacity.

**Table 2:** Clinical investigations of gut microbiota’s influence on osteoporosis therapy.

Key Subjects	Study Concept	Treatment	Main Study Statements	Year of Publication	Reference
Traditional Chinese medicine (TCM)	N = 43 OP-patients (71–87 years)	0.5 µg α-Calcitol as a capsule with or without the combination of Yigu decoction from TCM/day for 3 months	A positive change in the composition of the intestinal microbiome; An improved BMD, which is associated with a better regeneration of OP patients.	2023	[162]
Menopause, phytoestrogens	N = 100 post-menopausal, osteopenic women (50–85 years), double-blind, placebo-controlled trial	Calcium/vitamin D3 capsules and Lifenol^®^ hop extract or placebo/day for 48 weeks	Phytoestrognic Lifenol^®^-treatment was associated with an increase in intestinal *Turicibacter* and *Shigella* proportion as well as BMD.	2023	[163]
Menopause, probiotics	N = 40 post-menopausal women with OP, double-blind, placebo-controlled trial	Calcium, calcitriol and *Bifidobacterium animalis subsp. lactis* Probio-M8 or placebo/day for 3 months	↓ Bone loss ↑ Osteoblast activity ↑ Vitamin D3 level ↓ PTH and procalcitonin levels in serum ↓ ALP ↑ GM interactive correlation network A probiotic co-therapy promoted vitamin D3 levels as well as the microbiotic network of the intestine. ↓ procalcitonin.	2023	[23]
Menopause, inflammation	N = 20 post-menopausal, osteopenic women, double-blind, placebo-controlled trial	*Lactobacillus reuteri* or placebo/day for 12 months	Improved the microbiota composition as well as biofilm formation. Inflammatory parameters were reduced.	2022	[156]
Early postmenopausal women	N = 249 early postmenopausal women aged 59·1 (3·8) years randomized, double-blind, placebo-controlled trial	Daily probiotic with *L. paracasei* DSM 13434 + *L. plantarum* DSM 15312 + *L. plantarum* DSM 15313 for 1 year	↓ LS-BMD loss	2019	[20]
Postmenopausal women	N = 90 women, aged 75–80 years randomized, double-blind, placebo-controlled trial	*L. reuteri* 6475	↓ Loss of total vBMD	2018	[157]
Postmenopausal women	N = 67 healthy women. double-blind, placebo-controlled trial	*Bacillus subtilis* C-3102	↑ BMD ↓ Bone resorption ↓ uNTx ↓ TRACP-5b ↑ *Bifidobacterium* ↓ *Fusobacterium*	2018	[158]
Menopause, phytoderivates, prebiotics	N = 78 osteopenic, post-menopausal women, randomized, placebo-controlled study	Calcium, calcitriol, magnesium with 60 mg isoflavone aglycones plus probiotics or placebo/day for 12 months	↑ Bone turnover, Modulated estrogen metabolism. ↓ BMD loss.	2017	[159]
Adolescence, probiotics	N = 28 healthy, adolescent women (11–14 years), randomized crossover study	0, 10, or 20 g soluble corn fiber/day for 4 weeks	↑ GM composition ↑ Calcium absorption.	2016	[160]
Menopause, phytotherapeutics	N = 34 post-menopausal, healthy women, randomized crossover study	37mg isoflavone and 5g fructooligosaccharides or placebo/day for 2 weeks	After these interventions, no significant changes in the GM were observed.	2013	[161]

Abbreviations: BMD—bone mineral density, GM—gut microbiota, OP—osteoporosis, TCM—traditional Chinese medicine. The up arrow (↑) indicates activation/increase/high regulation and the down arrow (↓) indicates decrease/decrease/regulation/suppression.

### 3.5. Resveratrol

Resveratrol is a natural polyphenol belonging to the stilbene group and has been shown to have a variety of effects, especially regenerative and cardio-protective effects as well as properties to modulate the mechanisms of tumorigenesis. including epithelial–mesenchymal transition [164], obesity [26], and a variety of aging processes [165]. In addition, an important function of polyphenols, such as resveratrol, is now being increasingly recognized in relation to gut health, including stabilizing the gut barrier and promoting a wide range of beneficial GM [166]. Interestingly, resveratrol was discovered as early as 1939/40 [167], but its pharmaceutical prophylactic and adjuvant use still remains relatively undiscovered among clinical practitioners due to the continued lack of clinical trials and is therefore rarely used in clinical practice, especially in European countries. However, the French paradox, which refers to the cardio-prophylactic effect of consuming red wine containing resveratrol while consuming a diet high in saturated fat, was one of the first discoveries and most well-known effects [168]. Considering that the natural poly-phenol has been found in a large number of natural products such as fruits, herbs, seeds, and nuts, resveratrol is characterized by no adverse side effects when used in moderate doses, confirmed by a clinical trial over 2 years [169]. The phytoalexin has been found to occur in the form of two isomers, with the trans form being more abundant due to its greater molecular stability compared to the cis form [28]. 

A generally recognized limiting factor of oral resveratrol is its low bioavailability of less than 1%, although the absorption rate by transepithelial diffusion is about 75% [170]. However, there is increasing evidence that resveratrol is metabolized in the liver, and gut cells and by beneficial GM into various metabolites that have recently been shown to be even more potent than unmetabolized resveratrol, which must be taken into account when evaluating its pharmacokinetics [30]. In this regard, results have already been published on more sensitive analytical assays that quantify more accurately resveratrol metabolites, including those produced by GM, and thus better assess the metabolic patterns of resveratrol [171]. 

In light of the fact that GM-derived resveratrol metabolites such as lunularin and dihydroresveratrol show enhanced beneficial bioactivity over resveratrol, the significance of the interaction between polyphenols and GM for organ health, including bone health becomes evident [30]. In parallel, the findings that resveratrol acts as a prebiotic by promoting the growth and activity of beneficial bacteria including SCFA-producing species [172], which may in turn produce more multifunctional anti-inflammatory products such as SCFA, known to actively modulate bone remodeling [8], underscore the need to understand the fundamental principles of OP development. Of note, the anti-inflammatory and anti-oxidant function of the Sirt-1 activator via central signaling pathways, including the modulation of pro-inflammatory signaling pathways, such as NF-κB, is widely recognized in basic research and has been demonstrated in various models of chronic inflammatory diseases, such as OP and cancer [165]. This increasingly extends to additional models where the regulation of GM plays a central role [173]. 

Therefore, the interaction of resveratrol and the GM, particularly in relation to bone health, will be illuminated from multiple perspectives and discussed regarding a possible future preventive and adjuvant therapy option. In this context, resveratrol is also discussed as a phytoestrogen [174] including its unique effects on the extracellular matrix (ECM) at the epigenetic level by modulating estrogen receptor activity and stimulating Runx2 expression through the Sirt-1 axis in bone tissue [24]. Additionally, it acts as an epigenetic immune modulator in conjunction with the GM [175].

#### 3.5.1. Resveratrol-Microbiota-Axis

**Figure 4:**
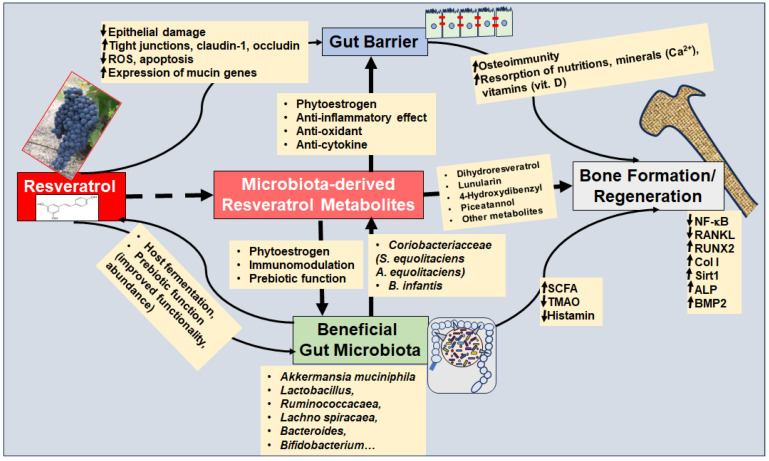
The resveratrol–microbiota axis modulates bone remodeling in primary and secondary osteoporosis. The up arrow (↑) indicates up-regulation and the down arrow (↓) indicates suppression. Abbreviations: A. equolifaciens—Adlercreutzia equolifaciens; ALP—alkaline phosphatase; B. infantis—Bifidobacterium infantis; BMP—bone morphogenetic protein; Col I—Collagen I; NF-κB—nuclear factor-kappa B; RANKL—receptor activator of NF-κB ligand; ROS—reactive oxygen species; Runx2—Runt-related transcription factor 2; SCFA—short chain fatty acids; S. equolifaciens—Slackia equolifaciens; Sirt1—Sirtuin 1; TMAO—Trimethylamine N-oxide.

Before discussing resveratrol as a prebiotic, its multifunctional regenerative strengthening of the gut barrier will be considered, which is an integrative prerequisite for several essential factors, including an intact immune system, prevention of the passage of GM-produced endotoxins and other pro-inflammatory mediators into circulation, as well as intact absorption of nutrients, vitamins, and minerals (e.g., calcium). To the author’s knowledge, there are no current studies on the resveratrol–microbiota axis regarding OP. Therefore, the following sections present findings on the mode of action of resveratrol and GM in general and derive a hypothetical functioning in relation to bone metabolism (Figure 4).

##### Resveratrol Restores Gut Barrier

The intestinal barrier is essentially formed by transmembrane proteins, referred to as tight junctions, which ensure that the membranes of the epithelial cells in the intestine lie close together and thus form a barrier for the intestinal mucosa. An important finding is that tight junction proteins including claudin-1, occludin, and Zonula occludens-1 (ZO-1) have been found to be reduced in several types of OP, such as glucocorticoid-induced OP [176] and age-related OP [177]. Interestingly, resveratrol has been found to stimulate the expression of claudin-1, occludin, and ZO-1 in intestinal epithelial cells via modulation of the PI3K/Akt-mediated Nuclear factor erythroid-2-related factor 2 (Nrf2) signaling pathway [178] (Figure 4). In this context, increased anti-oxidative processes, reduced intracellular reactive oxygen species (ROS) levels and decreased rates of apoptosis by resveratrol have been demonstrated [178] (Figure 4). 

In addition, resveratrol was found to stimulate regeneration of LPS-shrunken or disrupted microvilli in the ileum through modulation of NF-κB signaling and Nrf2 up-regulation, which has also been shown in correlation with aforementioned restoration of LPS-damaged tight junctions [179]. The integrity of the microvilli in the intestine is important because they ensure the cleaning as well as the enlargement of the intestinal surface, which is an essential prerequisite for the metabolism of compounds. The absorption of nutrients and minerals, including calcium, thus ensuring the effectiveness of OP therapies. The positive effect of resveratrol on microvilli was confirmed by the results of intra-amniotic administration of resveratrol in the absence of external factors such as endotoxins [180]. In fact, the number and morphology of globular cells were also modulated by resveratrol, which supports its positive effect on the intestinal barrier [180]. 

Probiotics with *Lactobacilli* have shown a clinically beneficial effect on preventing or alleviating bone loss that correlates with restoration of the gut barrier, emphasizing the significance of intact intestinal tight junctions [176,177]. Interestingly, resveratrol has been shown to protect *Lactobacillus* reuteri from oxidative stress by modulating the expression of enzymes including oxidoreductase, thereby further indirectly modulating/supporting the intactness/integrity of the gut barrier [181] (Figure 4, Table 3). The improved functionality [182] and abundance of various *Lactobacillus* species due to resveratrol [183,184] have been confirmed by other studies (Table 3). Evidence suggests that resveratrol is multifunctional in strengthening the gut barrier, as the natural polyphenol affects the gut barrier through numerous other pathways, such as indirect modulation of the endocannabinoid system, which has been shown to correlate with the promotion of beneficial GM including *Ruminococcacaea* and *Akkermansia Muciniphila* [185] (Figure 4, Table 3). Importantly, *Akkermansia Muciniphila* has been found to synergistically improve gut homeostasis by secreting Wnt/β-Catenin-activating proteins leading to enhanced proliferation and regeneration of gut stem cells [186], which is a direct reflection of the synergistic interaction between GM and host.

Overall, it can be concluded that resveratrol has a multifunctional synergistic effect in terms of strengthening the gut barrier, both directly through its protective effect on the gut epithelial cells and indirectly through the modulation of GM composition, which suggests a prebiotic property of resveratrol, which is focused on within the next subsection.

#### 3.5.2. Resveratrol as a Prebiotic Active Agent

Dysbiosis has been postulated as an important driving factor for intestinal mucosal inflammation associated with dysregulated intestinal immune cells, which is crucial for the development of chronic inflammatory diseases including OP [187]. Therefore, targeting dysbiosis by promoting beneficial GM and suppressing harmful species is of particular interest as an integral treatment regime for OP. In addition to probiotics referring to GM intake, sufficient intake of prebiotics is a necessary prerequisite for the effect of probiotics and the long-term maintenance of a healthy GM composition. Prebiotics are defined as substrates that are fermented by beneficial GM, promoting the growth of specific GM species and also improving GM activity [188]. Most common prebiotics refer to indigestible carbohydrates that specifically show the aforementioned prebiotic characteristics without further bioactive properties [188]. Interestingly, stilbenoids such as resveratrol have also been found to be anaerobically fermented by GM and modulate GM composition including *Firmicutes/Bacteroidetes* ratio and promotion of beneficial species including *Faecalibacterium prausnitzii* [189] (Figure 4). Preclinically, resveratrol has been found to promote growth performance, intestinal morphology, and GM composition and metabolism in correlation with modulation of pro-inflammatory interleukins and stimulation of SCFA production [172]. 

A reduction of beneficial SCFA-producing GM is associated with suppressed osteogenesis [4] consistent with SCFA stimulating BMP signaling by inhibiting histone deacetylation [190]. In terms of modulating dysbiosis, resveratrol reversed dysbiosis after a high-fat-diet, reflected in a greater abundance of beneficial species such as *Allobaculum*, *Bacteroides*, and *Blautia*, known as SCFA-producers [191] (Table 3). This is in accordance with findings that GM modulation by resveratrol correlates with increased butyrate levels, suggesting that beneficial SCFA-producing GM were promoted by resveratrol [192]. Evidence further suggests that improved diversity of the GM by resveratrol correlates with modulated oxidative stress, linked with suppression of harmful species [193] (Table 3). Other findings support the promotion of beneficial GM including *Lactobacillus* and *Bifidobacterium* in correlation with decreased TMAO levels by resveratrol [194] (Table 3). 

In addition, resveratrol was shown to positively modulate the activity of GM epigenetically, such as enhancing the adhesion of *Lactobacillus acidophilus* by modulating its surface layer proteins, with four up-regulated and twelve down-regulated proteins [195] (Table 3). It is noteworthy that resveratrol showed the highest adhesive effect on *Lactobacillus acidophilus* compared to other polyphenols, including epicatechin, caffeic acid, and hesperidin [195]. However, other results suggest that the combination of resveratrol and other polyphenols such as quercetin combined with a probiotic shows a positive synergistic effect on GM composition and metabolism including metabolite production and anti-oxidant activity [183]. 

As a recognized phytoalexin, resveratrol further demonstrates bacteriostatic and/or bactericidal effects on harmful GM species including viruses and fungi [196]. In this context, various mechanisms of resveratrol have been identified, such as the modulation of motility and flagellar genes in *E. coli* or the modulation of toxins, including the cholera toxin of *V. cholerae*, or the modulation of hemolysis of *Staphylococcus* (*S.*) *aureus* [196]. Consequently, resveratrol has been shown to synergistically complement various conventional standard antibiotics while mitigating undesirable side effects [196], as confirmed by preclinical in vivo results regarding the prophylaxis of campylobacteriosis [197]. Interestingly, resveratrol metabolites such as picetannol have also been found to have antimicrobial activity, including efficacy against *S. aureus* [198]. It is becoming increasingly recognizable that the GM is a critical interface for the optimal efficacy of resveratrol, as resveratrol metabolites are not only metabolized by the liver but also by the GM, which is explained in more detail in the next chapter.

**Table 3:** Resveratrol’s effect on gut microbiota.

Study Design	Resveratrol Treatment	Modulatory Effect on GM	Year of Publication	Reference
In vitro *L. acidophilus* NCFM (Danisco)	0.5 mM resveratrol	↑ adhesion of *Lactobacillus acidophilus* Modulation of surface layer proteins. Resveratrol demonstrated the most significant effects when compared to other polyphenols such as epicatechin.	2022	[195]
In vivo Male Five-week-old C57BL/6 J mice; standard specific-pathogen-free facility; high-fat diet	Oral resveratrol (300 mg/kg/day)	Strengthened intestinal barrier by decreasing the uptake of FITC-dextran and LPS levels. ↑ *Parabacteroides* (*Anaerotruncus*, *Oscillibacter*, *Romboutsis*) ↓ *Lachnospiraceae*, *Coprococcus1*, *Roseburia*, *Desulfovibrio*	2022	[191]
in vitro colonic fermentation *Limosilactobacillus fermentum*	*Limosilactobacillus fermentum* (160 mg), quercetin, and or resveratrol (150 mg)	↑ *Lactobacillus* spp., *Enterococcus* spp. and *Bifidobacterium* spp. ↓ *Bacteroides* spp./*Prevotella* spp., *Clostridium histolyticum*, *E. rectale*/*C. coccoides* ↑ anti-oxidant activity within the gut	2022	[183]
In vivo Female C57BL/6 mice (20–25 g, 8–12 weeks, specific pathogen-free)	200 mg/kg/day resveratrol for 14 days intragastrically	Beneficial impacts on GM composition. ↑ butyrate levels	2022	[192]
In vivo Three-week-old C57BL/6 mice	resveratrol 10/20/50 mg/kg/day for 4 weeks	↑ *Butyricicoccus*, *Ruminococcus 1*, and *Roseburia* ↑ amino acids/lipid metabolism, defense mechanisms of GM) ↓ expression of IL-6 and IL-1β ↑ expression of propionic-, isobutyric-, butyric-, and isovaleric acid	2021	[172]
In vivo Male Sprague–Dawley (SD) rats	Oral resveratrol of 100 mg/kg·bw/day for 6 weeks	↑ gut barrier, ↑mRNA levels of occludin, Zo-1, claudin1, modulation of endocannabinoid. ↑ *Ruminococcacaea*, *Akkermansia Muciniphila*, *Lachnospiraceae* ↓ Desulfovibrio	2020	[185]
In vivo Six-week-old C57BL/6 J male mice	300 mg/kg/day trans-resveratrol	↓ *Bacteroides*, *Desulfovibrionaceaesp*	2020	[173]
In vivo 30 five-week-old male Wistar rats under a high-fat diet	400 mg/kg resveratrol, 200 mg/kg sinapic acid or 400 mg/kg resveratrol and 200 mg/kg sinapic acid for 8 weeks	↑ *Lachaospiraceae* (*Blautia* and *Dorea*) ↓ *Bacteroides* and *Desulfovibrion-aceaesp*. ↓ Oxidative stress correlated with higher diversity in GM.	2019	[193]
In vivo Female BALB/c mice (aged 6–8 weeks	Resveratrol was given 24 h prior to TNBS injection and given daily for 5 days	↑ *Rinococcus gnavus* and *Akkermansia mucinphilia*; ↓ Th1/Th17 cells; ↓ *Bacteroides acidifaciens*	2019	[175]
*Lactobacillus reuteri* PL503 in MRS broth	Resveratrol (100 μM)	↑ antioxidant functions of *Lactobacillus reuteri* protect it from oxidative stress by H_2_O_2_. ↑ dhaT gene	2019	[181]
In vitro Six *Lactobacillus* strains	Quercetin/resveratrol concentrations of 2048 and 1400 μg/mL	Quercetin and resveratrol combined with *Lactobacillus probiotics* might enhance their impact in the host.	2019	[182]
In vivo Female C57BL/6J mice and ApoE^−^/^−^ mice with a C57BL/6 genetic background	0.4% resveratrol for 4 months	↑ *Lactobacillus, Bifidobacterium*; ↓ TMAO levels	2016	[194]
In vivo Male Kunming mice under high fat diet	200 mg resveratrol per kg per day for 12 weeks	↑ ratio of *Bacteroides* and *Firmicutes* ↑ *Lactobacillus, Bifidobacterium*; ↓ *Enterococcus faecalis*	2014	[184]

Abbreviations: GM—gut microbiota, spp.—abbreviation for more than one bacteria species, TMAO—Trimethylamine N-oxide, TNBS—2,4,6-trinitrobenzene sulfonic acid, mRNA—messenger RiboNucleic Acid, ZO-1—Zonula occludens-1, FITC—Fluorescein isothiocyanate, LPS—Lipopolysaccharide, Th1—Type 1 T helper, Th17—T helper 17, IL—interleukin, E. rectale—Eubacterium rectale, C. coccoides—Clostridium coccoides. The up arrow (↑) indicates activation/increase/high regulation and the down arrow (↓) indicates decrease/decrease/regulation/suppression.

#### 3.5.3. Gut Microbiota Derived Resveratrol Metabolites

A growing body of evidence suggests that GM plays a crucial role in the metabolization of resveratrol and its precursor piceid into a number of resveratrol metabolites [30,199] (Table 4). The conversion of piceid to resveratrol has been shown by several *Bifidobacteria* and *Lactobacillus* spp. through the expression of β-glucosidase, highlighting the importance of these species within the human gut [199]. Of high clinical interest, the activity of β-glucosidase has been found to differ significantly between these species, whereby *B. infantis* was particularly found with a very fast rate of piceid conversion [199], hypothesizing its particular importance for human health. Remarkably, piceid showed no measurable/discernible anti-inflammatory effects compared to resveratrol [199], highlighting the importance of its metabolization. This is particularly noteworthy considering that piceid is two to three times more abundant than trans-resveratrol in many natural foods, including red grape juice, cocoa powder, and red wine [200].

Also, with regard to resveratrol, its metabolization appears to be of major importance, as resveratrol metabolites such as lunularin and dihydroresveratrol exhibit greater bioactivity than unmetabolized resveratrol, including enhanced prebiotic [201], anti-inflammatory and anti-cancer effects [30] (Table 3, Figure 4). Of note, only specific bacteria species have been found with the ability to further metabolize resveratrol including *Coriobacteriaceae* such as *Slackia equolifaciens* and *Adlercreutzia equolifaciens* [202]. This finding presents a significant argument for elucidating the varying outcomes observed in clinical studies involving resveratrol, as differences in GM composition among patients or healthy volunteers may influence the metabolism of both piceid and resveratrol. Specifically, the presence or absence of the aforementioned species capable of metabolizing piceid and resveratrol could contribute to observed discrepancies [202]. Indeed, a lower bacterial sequence richness from *Bateroidetes*, *Actinobacteria*, *Verrucomicrobia* and *Cyanobacteria* was associated with a lack of dihydroresveratrol production, although trans-resveratrol was supplied [202]. *Eggerthella lenta*, which belongs to the phylum *Actinobacteria*, promoted the efficacy of resveratrol through reductive metabolism correlated with significant transcriptional changes of 491 genes, where 283 genes were up-regulated [203]. In addition, eleven other bacteria, including *B. cereus*, *A. denitrificans*, and *E. coli*, were found to convert about 20 percent of resveratrol into resveratroloside, dihydroresveratrol, and other resveratrol metabolites [204] (Table 4). Additional findings supported the metabolization of resveratrol by *E. coli* expressing the resveratrol reductase gene LUA64_RS01375 [203].

Of note, in the previous chapter, the positive effect of resveratrol on *lactobacilli* was described, but this could also be based on resveratrol metabolites. For instance, resveratrol-3-O-sulfate caused a 10-fold increase in the growth of *Lactobacillus reuteri*, whereas resveratrol showed no effects [166] (Table 4). The prebiotic effect of resveratrol 3-O-sulfate correlated with an up-regulation of mRNA expressions of tight junction and mucin-related proteins [166]. Additional study findings suggest a prebiotic effect of resveratrol metabolites, as evidenced by an observed enhanced synergistic effect on *Ligilactobacillus salivarius* by dihydroresveratrol [201] (Table 4). 

Although limited research is available to confirm the positive direct effects of resveratrol metabolites on regenerating bone tissue, preliminary evidence suggests that piceatannol (3,3′,4,5′-tetrahydroxy-trans-stilbene) epigenetically promotes osteoblast formation by increasing BMP-2 levels [205] (Table 4). 

**Table 4:** GM-derived resveratrol metabolites and their multifunctional effects.

Resveratrol Metabolite	Study Design	Resveratrol/Resveratrol Metabolite Treatment	Prebiotic Effect or Functioning	Reference
Resveratrol-3-O-sulfate	In vivo HPLC-MS/MS In vitro Caco-2 cells	oral ingestion of 50 mg/kg resveratrol	↑ *Lactobacillus reuteri* ↑ expression of tight junction and mucin-related proteins	[166]
Dihydroresveratrol	In vivo specific pathogen-free C57/BL6 mice (5-week-old, male)	30 mg resveratrol or *Ligilactobacillus salivarius* every day on weeks 2, 3, 5, and 6	↑ improved synergistic effect of *Ligilactobacillus salivarius*	[201]
Fermented resveratrol	Fecal samples originated from four volunteer donors who were two males and two females	10 mg/mL resveratrol	↑ *Faecalibacterium prausnitzii*	[189]
Dihydroresveratrol and Lunularin	Male CD-1 mice (6 weeks), resveratrol for 4 weeks	diet with 0.05% resveratrol (4.6 mg/kg/day) or 0.025% dietary resveratrol	↑ anti-inflammatory and anti-cancer properties	[30]
Piceid (synonyms: resveratrol-3-O-β-d glucoside, polydatin)	Cell extracts of *Bifidobacterium* and *Lactobacillus* spp. (*B. infantis*, *B. bifidum*, *L. acidophilus*, *L. casei*, and *L. plantarum*)	200 μL piceid in 50 mM	Piceid-metabolization to resveratrol by *B. infantis*, *B. bifidum*, *L. acidophilus*, *L. casei*, and *L. plantarum* ↑ β-glucosidase activity for *B. infantis*; ↓ IL-6 and TNFα ↑ IL-10	[199]
Piceatannol (3,3′,4,5′-tetrahydroxy-trans-stilbene)	Immortalized fetal osteoblasts (hFOB), and osteosarcoma cells (MG-63)	piceatannol 2 mg/mL	↑ osteoblastogenesis ↑ BMP-2	[205]
Resveratroloside, piceid, and dihydroresveratrol and others	43 bacterial strains that are usually animal- or human-associated	50 μg resveratrol	Among the 43 bacteria tested, eleven had the ability to convert 20% of the resveratrol, including *B. cereus NCTR-466*, *A. denitrificans NCTR-774*, and *E. coli ATCC 47004*	[204]
Dihydroresveratrol	*Eggerthella lenta*	resveratrol 200 μM	↑ Resveratrol effectiveness by *Eggerthella lenta*. Modulated gene expression with 283 genes showing increased activity. ↑ IL-22 and IL-17A in the colon of mice.	[203]

Abbreviations: *A*.—*Achromobacter*, *B*.—*Bifidobacterium*, BMP—bone morphogenetic protein, *E. coli*—*Escherichia coli*, GM—gut microbiota, IL—interleukin, *L.*—*Lactobacillus*, TNF—tumor necrosis factor. The up arrow (↑) indicates activation/increase/high regulation and the down arrow (↓) indicates decrease/decrease/regulation/suppression.

#### 3.5.4. Resveratrol as a Phytoestrogen for Osteoporosis

The finding that estrogen deficiency indirectly triggers OP by favoring dysbiosis, associated with dysregulated immune cells and pro-inflammatory processes in the intestinal mucosa [206], is supported by findings that demonstrate the vital role of a healthy GM in maintaining bone health [53]. Therefore, natural phytoestrogens such as resveratrol offer opportunities to naturally compensate for estrogen deficiency by modulating the expression and activity of extra genital estrogen receptors (ERs), such as ERs within gut mucosa and bone tissue [207] (Figure 4). The great advantage of multifunctional active compounds such as resveratrol lies in their ability to modulate the receptors, meaning that they have both an agonist and antagonist effect depending on the prevailing microenvironment [174]. Since estrogen as a hormone has numerous mechanisms of action, including the stimulation of osteoblasts, the modulation of inflammatory pathways such as NF-κB and RANKL, and the maintenance of an intact immune system, which are all known as crucial factors for bone health, we present resveratrol accordingly. 

Resveratrol has been demonstrated to exert both estrogenic and estrogen-independent effects on bone cells [208]. Its phytoestrogenic property is supported by findings that resveratrol binds to ER-α of osteoblasts, activating signaling pathways that mimic those triggered by endogenous estrogen [174]. This activation leads to several down-stream effects that are beneficial for bone health. 

Firstly, resveratrol exerts direct effects on osteoblasts and osteoclasts, the two primary cell types involved in bone remodeling. In osteoblasts, resveratrol promotes cell proliferation and differentiation, leading to increased bone formation and repair. Mechanistically, resveratrol increases the expression of osteogenic markers associated with osteoblastic cell differentiation, proliferation and function, such as osteocalcin (OCN), osteopontin (OPN), bone sialoprotein (BSP), osterix, calcineurin, Runx2, bone morphogenetic protein-2 (BMP)-2, BMP-7, vascular endothelial growth factor (VEGF) and α1 type I collagen (COL1A1) [174,209]. Several studies have found that resveratrol enhances the formation of vascular tissue and bone both in vitro [210,211] and in animal models of OP, such as ovariectomized rodents, which mimic postmenopausal OP resulting from estrogen deficiency, as well as in aged animals [44,212]. Its phytoestrogenic function is supported by selective binding to ER-α of osteoblasts [213]. Resveratrol also increased alkaline ALP activity, collagen synthesis, and calcium deposition in osteoblast cells [209]. Interestingly, via ER-dependent extracellular signal-regulated kinase 1/2 and p38/MAPK activation [214] as well as via the Wnt/β-catenin signaling pathway, a key mediator of osteoblastogenesis [215], resveratrol was shown to enhance proliferation and osteoblastic differentiation in bone marrow mesenchymal stem cells (BMSCs), adult stem cells found in the bone marrow capable of differentiating into various cell types such as osteoblasts, chondrocytes, and adipocytes. Furthermore, resveratrol increases the expression of the osteogenic transcription factor core-binding factor-α1, promoting osteogenesis in bone-derived cells [42].

Secondly, resveratrol inhibits osteoclastogenesis, whereby precursor cells differentiate into osteoclasts, the cells responsible for bone resorption. By reducing osteoclast formation and activity, resveratrol helps to prevent excessive bone loss and maintain bone mass [174,209]. Concretely, resveratrol inhibits osteoclast formation and activity by suppressing the expression of key osteoclastogenic factors, such as RANKL, which is produced by osteoblasts and bone marrow stromal cells and binds to its RANK receptor on the osteoclast surface, activating central signaling pathways such as the NF-κB pathway [44,212]. In particular, our group demonstrated that resveratrol inhibits RANKL-induced NF-κB activation by targeting inhibitors of nuclear factor-kappa B alpha (IκBα) kinase. By blocking the activation of this enzyme, which regulates NF-κB activation by phosphorylating and degrading IκBα, resveratrol effectively prevented osteoclastogenesis in bone-derived cell lines [42]. On the contrary, osteoprotegerin (OPG), a soluble RANKL decoy receptor, antagonizes RANKL-mediated osteoclastogenesis by binding to RANKL, thereby obstructing its binding to the RANK receptor and consequently reducing bone resorption. Hence, maintaining a balanced RANKL/OPG ratio is fundamental to healthy bone metabolism. Khera et al. showed that resveratrol restores a balanced RANKL/OPG ratio in postmenopausal OP rat models [216]. Furthermore, RANKL/RANK/NF-κB signaling activation stimulates osteoclasts to synthesize and secrete catabolic enzymes such as matrix metalloproteinase (MMP) that resorb the bone ECM [42,217]. Importantly, exposure to resveratrol has been shown to elevate the levels of key ECM molecules, including collagen type I, collagen type II, β1-integrin, and osteocalcin, in cell cultures derived from bone tissue [218]. Briefly summarized, resveratrol exerts direct effects on osteoblasts and osteoclasts, the two primary cell types involved in bone remodeling.

Autophagy, the cellular process responsible for the degradation and recycling of damaged organelles and proteins, plays a crucial role in maintaining skeletal homeostasis, bone remodeling, and cartilage homeostasis. In the context of OP, dysregulation of autophagy has been implicated in the pathogenesis of this disease [219]. Reduced autophagic activity has been observed in osteoporotic bone tissues, leading to impaired osteoblast function, increased osteoclastogenesis, and, ultimately, bone loss [220]. Crucially, resveratrol activates autophagy in osteoblasts and inhibits it in osteoclasts, highlighting its impact on bone formation and resorption. Resveratrol modulation of autophagy was dose-dependent and reversible, indicating a finely tuned regulatory process [44]. By increasing the expression of autophagy-related proteins Beclin1 and LC3, and decreasing the expression of p62, resveratrol induced the osteogenic differentiation of pre-osteogenic MC3T3-E1 mouse cells [221] and significantly improved bone quality in osteoporotic rats [222]. Yang et al. demonstrated that resveratrol enhances mitophagy, a selective degradation process of damaged or dysfunctional mitochondria within cells [223], through the activation of the Sirt-1/PI3K/AKT/mTOR pathway [222]. In a similar fashion, resveratrol activated the AMPK/JNK1 pathway in ovariectomized rats, leading to the induction of autophagy and inhibition of apoptosis in osteocytes [224]. Osteocytes play an indirect yet pivotal role in bone remodeling by regulating the activity of osteoblasts and osteoclasts through the secretion and expression of specific signaling molecules, such as sclerostin, which inhibits the Wnt signaling pathway [225]. Of note, resveratrol protects osteocytes against oxidative stress-induced apoptosis through activating autophagy in osteocyte-like cells [224].

Chronic inflammation is involved in the pathogenesis of OP since it contributes to increased bone resorption and impaired bone formation [15], whereby dysbiosis associated with an inflamed intestinal mucosa, characterized by the loss of tight junctions, is the main factor, as discussed in the previous chapter. Resveratrol exercises potent anti-inflammatory effects by modulating various inflammatory pathways and signaling molecules within the bone microenvironment. For instance, it suppresses the activation of NF-κB, a key regulator of inflammation, thereby reducing the expression of inflammatory genes involved in bone resorption [28,42]. Moreover, resveratrol inhibits the expression of pro-inflammatory cytokines, such as IL-23, IL-17A, IL-6, TNF-α, and IL-1β, thereby promoting a slight increase in bone mineral density [216]. In fact, TNF-α and IL-1β have been shown to mediate cartilage degradation and apoptosis in chondrocytes [226]. Furthermore, several studies have demonstrated that OP increases the severity of cartilage damage that correlates with bone loss in osteoarthritis animal models [227]. Within this context, our group demonstrated that resveratrol exhibits chondroprotective properties by inhibiting IL-1β, thereby preventing the induction of chondrocyte apoptosis in vitro [218,228]. Similarly, we showed that resveratrol and curcumin, a polyphenol found in turmeric, exhibit synergistic effects in inhibiting IL-1β-induced NF-κB-mediated inflammation and apoptosis in chondrocytes. Moreover, this combination stimulates the expression of the cartilage-specific transcription factor Sox-9, which is crucial for cartilage ECM gene expression [229]. 

In addition to pro-inflammatory processes, oxidative stress, resulting from an imbalance between ROS production and anti-oxidant defenses, is involved in the pathophysiology of OP. Excessive ROS can promote osteoclastogenesis and osteoblast apoptosis, leading to increased bone resorption and decreased bone formation [230]. Resveratrol possesses potent anti-oxidant properties, acting as a scavenger of ROS and up-regulating endogenous anti-oxidant enzymes, such as superoxide dismutase (SOD) and catalase [231]. By reducing oxidative stress and protecting against ROS-induced damage, resveratrol helps to maintain bone health and prevent the development of OP [15]. Specifically, resveratrol inhibited the IL-1β-induced upregulation of ROS and induced ubiquitin-independent degradation of tumor suppressor protein p53 [228]. Moreover, resveratrol reduces RANKL-induced ROS production in murine osteoclast precursor RAW264.7 cells, likely through its anti-oxidant properties, leading to the inhibition of osteoclastogenesis [232]. 

Furthermore, compelling evidence indicates that resveratrol stimulates proliferation and osteoblastic differentiation in human and mouse MSCs [233]. In this context, our group found that TNF-β hampers the initial stages of bone cell formation in MSCs, however, resveratrol effectively reversed this negative impact of TNF-β on bone development by activating the Sirt1 and Runx2 pathways while also reducing NF-κB activation. These effects were consistent across both standard monolayer and high-density cultures of MSCs [24]. Similarly, Tseng et al. found that resveratrol enhanced osteogenesis while inhibiting adipogenesis in human MSCs by promoting the formation of the Sirt-1/FOXO3A transcriptional complex, which subsequently up-regulates the expression of Runx2 thereby inducing osteoblast-specific gene expression [234]. Taken together, these preclinical findings indicate a bone-protective role of resveratrol, support the translational potential of this phytoestrogen for the prevention and management of OP, and provide a basis for further clinical investigation. 

#### 3.5.5. Resveratrol as Epigenetic Modulator for Osteoporosis

Epigenetic modifications, including DNA methylation, histone modifications, and non-coding RNA-mediated gene regulation, play critical roles in modulating gene expression patterns without altering the underlying DNA sequence [235]. Dysregulation of these epigenetic processes is involved in various diseases, including cardiovascular, metabolic, and neurodegenerative disorders, cancer, and age-related diseases such as OP [236]. In recent years, accumulating evidence has suggested that resveratrol can exert its anti-oxidant, anti-inflammatory, anti-aging, anti-cancer, and anti-OP effects through epigenetic mechanisms and thereby influence gene expression patterns [237]. 

Histone modifications, including acetylation, methylation, phosphorylation, and ubiquitination, play critical roles in chromatin remodeling and gene expression regulation [68]. Resveratrol has been reported to modulate histone acetylation and methylation patterns by directly or indirectly affecting the activity of histone acetyltransferases histone deacetylases (HDACs), histone methyltransferases, and histone demethylases [237]. The Sirt proteins are members of the HDAC family. Sirts comprise seven HDACs known for their reliance on nicotinamide adenine dinucleotide for enzymatic activity. Through the deacetylation of both histone and non-histone proteins, such as transcription factors [238], Sirtuins are involved in a wide range of cellular processes, including aging, cardiovascular and metabolic disorders, cancer, and OP. Importantly, Sirt-1 regulates bone homeostasis by decreasing MSC differentiation into adipocytes while promoting differentiation into osteoblasts, which makes it a promising target for OP management [239]. Resveratrol, in turn, has been recognized as a potential regulator of Sirt-1 activity via epigenetic mechanisms [240]. For example, it has been demonstrated that resveratrol activates Sirt-1, which inhibits p300 acetyltransferase and reduces NF-κB-p65 acetylation, thereby inhibiting NF-κB transcriptional activation and osteoclastogenesis in bone-derived and preosteoblastic cells [42]. Similarly, by activating Sirt-1, resveratrol suppresses nicotinamide-induced adipogenesis while enhancing osteogenesis in MSCs, potentially through the deacetylation of Runx2 transcription factor in cultures of adipose-derived MSCs [25]. Taken together, all this preclinical evidence indicates that the interaction between resveratrol and Sirt-1 holds therapeutic potential for OP. Ultimately, the development of resveratrol-based interventions targeting Sirt-1 may offer new avenues for the prevention and adjunct treatment of primary and secondary OP, particularly in high-risk populations such as postmenopausal women, breast and prostate cancer patients, and the elderly. 

Non-coding RNAs, including miRNAs and long non-coding RNAs, play crucial roles in post-transcriptional gene regulation and chromatin remodeling [241]. A total of 331 miRNAs with altered expression were found in OP, with 122 miRNAs up-regulated, and 209 miRNAs down-regulated, linked to 105 target genes, which highlights miRNAs as interesting target molecules for OP prevention and adjunct treatment approach [242]. This is supported by the fact that miRNAs are key regulators of osteoblast differentiation in MSCs [243]. 

For instance, miR-181a-5p modulates inflammation and OP through the NF-κB/TLR pathway [244], affecting osteoclast formation and bone resorption. Resveratrol increased miR-181a-5p expression in osteoclast precursor RAW264.7 cells, reducing their differentiation into osteoclasts and alleviating LPS-stimulated OP [245]. miR-338-3p has consistently been shown to help prevent OP by targeting multiple signaling molecules involved in bone remodeling, including Runx2, fibroblast growth factor receptor 2, and RANKL and proprotein convertase subtilisin/kexin type5 [246]. Along these lines, Guo et al. demonstrated that resveratrol treatment down-regulated miR-338-3p expression, leading to increased expression of Runx2 in human osteoblast cells [247]. Resveratrol also suppressed the activation of the NADPH oxidase-4/NF-κB signaling pathway and the expression of cathepsin K, a lysosomal enzyme involved in bone matrix degradation, through increased miR-92b-3p expression, leading to a decrease in OP induced by estrogen deficiency in ovariectomized rats [248]. Finally, miR-193a down-regulation promotes osteogenic differentiation in BMSCs [249]. Song et al. demonstrated that resveratrol enhances SIRT-7/NF-κB activation via miR-193a inhibition, potentially promoting osteogenic differentiation of BMSCs and mitigating OP in ovariectomized rats [250]. Interestingly, the fact that dysbiosis including an increased abundance of *Firmicutes*, *Klebsiella pneumonia*, *E.coli*, and *Shigella*, has been shown to correlate with altered miRNAs associated with OP, further highlights resveratrol’s potential indirect epigenetic influence on bone remodeling [121].

In summary, these findings indicate that resveratrol exerts its biological effects through multifaceted interactions with epigenetic mechanisms, including DNA methylation, histone modifications, and non-coding RNA-mediated gene regulation directly by targeting molecules such as Sirt-1 and indirectly via targeting GM and their epigenetic impact through SCFA production and influence on miRNA expression. However, further elucidation of the molecular mechanisms underlying resveratrol-mediated epigenetic modifications is warranted and may lead to the development of novel therapeutic strategies for OP. Additionally, exploring combinatorial approaches involving resveratrol and other epigenetic modulators may offer synergistic therapeutic benefits for bone disorders.

## 4. Clinical Evidence

Natural polyphenols such as resveratrol are increasingly recognized in clinical OP prophylaxis and co-treatment, as evidenced by improved bone regeneration along with a variety of other beneficial regenerative processes such as improved gut barrier function as well as the prebiotic effects of oral and gut microbiota. To the authors’ knowledge, there is currently no clinical trial that includes the concept of simultaneous modulation of dysbiosis and regeneration of the intestinal mucosa and bone tissue by using resveratrol in OP patients. However, various clinical studies provide clinical evidence for a corresponding effect of the natural substance via the gut–bone axis, as demonstrated by improved bone regeneration through modulated osteogenic biomarkers [251], reduced osteoporotic fracture risk, and enhanced BMD [252]. The evidence for a modulation of the gut–bone axis comes primarily from population studies with specific dietary habits, such as the Western diet, a diet that correlates with dysbiosis and OP, whereas Mediterranean and anti-inflammatory diets are associated with healthier GM and reduced risk of OP [2]. The hypothesis is further supported by evidence that a polyphenol-rich diet with 1391 mg/day—including natural piceid and resveratrol from natural food such as cocoa powder and pomegranate juice –positively correlates with an increase in fiber-fermenting and butyrate-producing bacteria such as the family *Ruminococcaceae* and members of the genus *Faecalibacterium*, indicating prebiotic effects [32] (Table 5). Likewise, another study confirmed that the dietary intake of polyphenols from red wine modulates GM demonstrating an increased abundance of *Enterococcus*, *Prevotella*, *Bacteroides*, *Bifidobacterium*, *Bacteroides uniformis*, *Eggerthella lenta*, and *Blautia coccoides*, *Eubacterium rectale* and the inhibition of pro-inflammatory GM species [253] (Table 5). Concretely, increased SCFA-producing *Bifidobacteria* were linked to improved levels of cholesterol and C-reactive protein [253]. This is in accordance with a similar study showing that both dealcoholized and alcoholized red wine is associated with an increased abundance of fecal *Bifidobacteria*, *Lactobacillus* and *Faecalibacterium prausnitzii*, and *Roseburia* known to produce SCFA such as butyrate [254] (Table 5). It can be assumed that the promotion of *Bifidobacteria* by resveratrol further functions through beneficial cross-feeding mechanisms with species such as *Faecalibacterium prausnitzii*, which are thereby promoted [92]. The modulating prebiotic effect was further demonstrated by decreased LPS-producing GM species such as *Escherichia coli* and *Enterobacter cloacae* [254] (Table 5). Therefore, a diet rich in polyphenols is postulated to be an important co-therapeutic factor in the prevention and treatment of OP. Since piceid is the precursor of resveratrol, which has been shown to be metabolized to exert its beneficial effects (see Section 3.5.3), consumption of polyphenol-rich beverages and foods containing predominantly piceid may have differential effects in the absence of resveratrol-metabolizing GM species such as *B. infantis*. 

In practice, the implementation of a natural polyphenol-rich diet may present additional challenges, especially in OP patients with increased intestinal permeability, such as those with intestinal bowl diseases and diverse food intolerances, because many foods, especially histamine-containing foods, including red wine and various fruits and vegetables, may be temporarily intolerable [255]. This is of particular clinical significance because of the demonstrated correlation between increased intestinal permeability, estrogen deficiency, and low BMD [21]. Therefore, individualized resveratrol supplementation should also be considered, especially since resveratrol’s anti-inflammatory and anti-oxidant properties generally positively modulate the intestinal microenvironment [256]. Early clinical evidence confirms positive prebiotic effects such as an increase in *Akkermansia muciniphila* [257] (Table 5), known as a crucial bacteria for a healthy gut barrier [186]. Accordingly, a polyphenol-rich diet correlates with improved intestinal permeability as demonstrated by reduced serum zonulin levels among individuals aged ≥ 60 years, indicating an improved intestinal barrier [32]. 

In addition, maintaining an intact intestinal barrier and mucosa is particularly important in the prevention and treatment of OP, as an intact intestinal mucosa is necessary for calcium absorption. Interestingly, clinical evidence has already shown that individuals with pro-inflammatory lifestyle habits such as smoking and excessive alcohol consumption appear to particularly benefit from resveratrol, as evidenced by increased BMD following resveratrol supplementation, which also correlated with significantly improved calcium and vitamin D levels compared to control groups [258]. Although Bo et al. did not examine GM, including dysbiosis, in the subjects of this study, it can be hypothesized that the improved calcium absorption of the study participants is mainly associated with the anti-inflammatory effect of resveratrol on the intestinal mucosa, as well as the simultaneous prebiotic effect on GM species such as *Lactobacilli* [135,182]. Nevertheless, the complexity of the circumstances of the individual GM has likewise been demonstrated as differences between participants appeared to be linked to external environmental factors such as country of origin [257] (Table 5). In fact, this is largely to be expected as GM is recognized to be influenced by individual lifestyle factors, including cultural dietary habits [259], highlighting the need for individualized, personalized GM diagnostics before and during the treatment of dysbiosis by using resveratrol as an adjuvant treatment option. 

Importantly to note is the fact that resveratrol as a monotherapy cannot provide a short-term improvement in severe chronic diseases such as chronic kidney disease, which involves systemic high-grade inflammatory processes that also affect the gut mucosa and GM, especially if there is also an inadequate supply of fiber, minerals, and phytochemicals, as evidenced in a clinical trial [260]. However, it should not be assumed that resveratrol is ineffective. Rather, prospective studies should consider using an integrated therapy concept with resveratrol as a co-therapeutic agent alongside stress reduction through diet, physical exercise, and mental resilience in addition to conventional standard therapy. Vitamin D levels should also be measured, especially in OP patients with primary kidney disease, as the kidneys are essential for adequate vitamin D levels [261], which could also be one of the main causes of dysbiosis in addition to the inflammatory processes [262].

Another important factor to consider is that clinical trials need to measure both resveratrol and resveratrol metabolites, which can reveal potential biases such as differences in metabotypes, as demonstrated in clinical trials where 26% of healthy volunteers showed an inability to produce lunularin [263] (Table 5). Interestingly, the non-producer metabolic type was more common in women and independent of BMI and age [263], suggesting a relationship with female hormones. Other differences have been demonstrated within the lunularin metabotype population, such as the ability to metabolize lunularin to 4-hydroxydibenzyl [264] (Table 5). In light of the fact that 17 derivatives of resveratrol were found in blood plasma and urine samples after oral consumption of grape extract or moderate consumption of red wine, these findings support an increased persistence of polyphenols in the intestine [265]. These findings suggest underlying differences in GM composition, including resveratrol metabolizing GM species such as *B. infantis*, and resveratrol metabolic patterns have already been suggested as a useful marker of resveratrol efficacy in clinical and epidemiological studies [171]. Differences in individual GM composition, including polyphenol-metabolizing GM species, may further explain the current controversies surrounding resveratrol in clinical trials, also with respect to OP.

Resveratrol supplementation, either alone or in combination with phytoestrogens, has further shown promising results in increasing total body BMD and improving bone turnover parameters in postmenopausal women [266]. Notably, results from the randomized, placebo-controlled trial Resveratrol for Healthy Aging in Women (RESHAW) showed that resveratrol intake (75 mg twice a day) is correlated with a lower risk of hip fracture, particularly in women, and postmenopausal women had better BMD at the lumbar spine and femoral neck after supplementation [251]. Besides a diet rich in polyphenols, physical activity is known to be crucial to maintain balanced bone remodeling, and clinical trials combining resveratrol supplementation with regular physical activity have shown promising results for postmenopausal women with osteopenia, the elderly population as well as for patients with cancer-related OP [251,267,268]. Moreover, resveratrol has demonstrated benefits in individuals at risk for secondary OP, such as those with high alcohol consumption, obesity, or type 2 diabetes mellitus [251,258]. 

A further clinical rationale for the use of resveratrol in OP patients emerges from the double-edged sword of conventional pain management using pain killers such as NSAIDs, as there is evidence that NSAIDs have a negative effect on GM [16] and can also interfere with optimal osteogenic processes [269], highlighting the need for new co-therapeutic therapies. Resveratrol, which has already been clinically confirmed to reduce pain perception in postmenopausal women [169], is a promising approach, based on the confirmed multifunctional modulation of the NF-κB signaling pathway [42]. Although there is no clinical study to date on the concept of modulation of the GM-pain-axis by polyphenols such as resveratrol, initial preclinical evidence with resveratrol supports the hypothesis [270]. 

Furthermore, as a complementary therapy alongside conventional OP treatment, resveratrol seems to be a promising natural substance to prevent negative side effects from the therapy with bisphosphonates. Among the most serious side effects of bisphosphonate therapy is osteonecrosis of the jaw. A prospective study has shown that the prevalence of osteonecrosis of the jaw is related to oral dysbiosis, associated with a deficient innate immune response [271]. Compared to the control group, an increase in *Firmicutes* was observed in patients who received biphosphonates and in patients who developed osteonecrosis [271]. Interestingly, early clinical results suggest a beneficial modulation of the oral microbiome by resveratrol [272]. 

Finally, it is important to note that resveratrol supplementation has been well-tolerated without serious side effects over extended periods [169]. Therefore, resveratrol supplementation in moderate amounts alongside lifestyle factors such as physical activity [268] appears to be promising in both primary and secondary OP patients, primarily as OP prophylaxis and secondarily as a co-therapeutic approach within an integral treatment regimen. In the absence of intestinal barrier disorders, the best way to consume polyphenols such as resveratrol appears to be through the daily diet, as an optimal ratio of fiber, minerals, and phytochemicals suggests that they optimally nourish a healthy gut and thus maintain bone health by supporting a favorable gut–bone axis [273].

**Table 5:** Clinical investigations of resveratrol’s modulation of the gut microbiota and GM-derived resveratrol metabolites.

Key Subjects	Study Concept	Resveratrol Treatment	Main Study Statements	Year of Publication	Reference
Resveratrol metabolite Dihydroresveratrol Lunularin 4-hydroxydibenzyl (4HDB)	Healthy volunteers (N = 195)	150 mg of resveratrol in the evening for 7 days	Distribution of lunularin metabotypes: 74% producers, 26% non-producers. The non-producer metabotype is more prevalent in females, irrespective of BMI and age. 4-styrylphenol reductase converts stilbenes to dibenzyls in both metabotypes. No 4-dehydroxylation was observed in stilbenes or dibenzyls	2022	[263]
Resveratrol metabolite (4-Hydroxydibenzyl)	N = 59 Aged 34.2 ± 9.5 (18–52) years; BMI 23.0 ± 2.7	Daily 150 mg resveratrol for 7 days	Urinary detection of 4-Hydroxydibenzyl post-resveratrol intake clinically confirmed. In vitro fecal probe incubations validated the findings.	2022	[264]
Increased intestinal permeability, butyrate-producing bacteria	N = 51 participants (aged ≥60 years).	8-week diet with 1391 mg/day (polyphenols daily such as cocoa powder and pomegranate, which contain resveratrol)	↑ fiber-fermenting and butyrate-producing bacteria, e.g., *Ruminococcaceae* family and *Faecalibacterium* genus; ↓ serum zonulin levels, ↓ blood pressure	2021	[32]
Cardiovascular risk factors, Trimethylamine N-oxide (TMAO)	N = 20 healthy participants.	600 mg Taurisolo^®^ with 135.7 µg/g resveratrol daily, for 4 weeks	↓ TMAO Reduction of TMAO attributed to anti-oxidative and GM modulating effects.	2019	[273]
Prebiotic effects	N= 28 Obese men with metabolic syndrome (Aged 48 ± 9 years)	2 g oral Resveratrol per day for 35 days	↑ fecal *Akkermansia muciniphila* ↓ *Rikenellaceae*, *Ruminococcus*, *Oscillospira*, *Clostridium*, *Alistipes*, *Odoribacter*, and *Butyricimonas* ↑ *Gammaproteobacteria*, *Gemellaceae*, *Turicibacter*, and *Atopobium*.	2019	[257]
Prebiotic effects	N = 20 men aged 48 ± 2 years (range 45–50 years)	Daily oral grape extract (A) or red wine (B) containing trans-resveratrol 0.64 ± 0.04 (A), 2.49 ± 0.06 (B) mg; cis-resveratrol 0.98 ± 0.07 (A), 0.30 ± 0.01 (B) mg; trans-Piceid 3.30 ± 0.20 (A), 1.91 ± 0.01 (B) mg; cis-Piceid 1.39 ± 0.08 (A) 0.02 ± 0.004 (B) mg; total resveratrol (A) 6.30 ± 0.09, 4.72 ± 0.07 (B) mg	↑ fecal *Bifidobacteria*, *Lactobacillus*, *Faecalibacterium prausnitzii*, *Roseburia* ↓ *Escherichia coli* and *Enterobacter cloacae* ↓ metabolic syndrome markers in obese patients	2016	[254]
Metabolism	N = 12 healthy men (19–28 years); BMI between 20–26	0.5 mg trans-resveratrol/kg body weight	Lunularin and 3,4′-dihydroxy-trans-stilbene were identified as GM-derived trans-resveratrol metabolites. *Slackia equolifaciens* and *Adlercreutzia equolifaciens* were identified as producers of dihydro-resveratrol.	2013	[202]
Pharmacokinetic, metabolism	N = 10 Healthy men(24–35 years); BMI 24.9	Daily oral grape extract (A) or red wine (B) containing trans-resveratrol 0.64 ± 0.04 (A), 2.49 ± 0.06 (B) mg; cis-resveratrol 0.98 ± 0.07 (A), 0.30 ± 0.01 (B) mg; trans-Piceid 3.30 ± 0.20 (A), 1.91 ± 0.01 (B) mg; cis-Piceid 1.39 ± 0.08 (A) 0.02 ± 0.004 (B) mg; total resveratrol (A) 6.30 ± 0.09, 4.72 ± 0.07 (B) mg	Blood plasma and urine samples revealed 17 derivatives of resveratrol, stemming from oral consumption or GM. Capsule intake prolonged polyphenol presence in the gut and enhanced metabolizable intake.	2012	[265]
Prebiotic effects	N = 10 healthy male volunteers (randomized crossover controlled intervention study)	Daily de-alcoholized (A) or alcoholized red wine (B) containing trans-resveratrol 0.74 ± 0.06 (A), 0.79 ± 0.10 (B); cis-resveratrol 0.75 ± 0.04 (A), 0.76 ± 0.04 (B); trans-Piceid 2.86 ± 0.26 (A) 2.56 ± 0.31 (B); cis-Piceid 1.93 ± 0.24 (A), 2.10 ± 0.09 (B) mg/dose for 30 days	↑ *Enterococcus, Prevotella*, *Bacteroides*, *Bifidobacteriu*, *Bacteroides uniformis*, *Eggerthella lenta*, and *Blautia coccoides–Eubacterium rectale*. ↓ Systolic and diastolic blood pressure, triglyceride, total cholesterol, HDL cholesterol, and C-reactive protein concentrations. Changes in cholesterol and C-reactive protein concentrations correlated with modulated *Bifidobacteria* levels. ↓ pro-inflammatory GM.	2012	[253]

Abbreviations: BMI—body mass index, GM—gut microbiota, TMAO—Trimethylamine N-oxide. The up arrow (↑) indicates activation/increase/high regulation and the down arrow (↓) indicates decrease/decrease/regulation/suppression.

## 5. Conclusions

Resveratrol as a prophylactic and co-therapeutic option for primary and secondary OP is supported by clinical evidence that a natural polyphenol-rich diet modulates gut and oral microbiota, thereby modulating the gut–bone axis. Importantly, dysbiosis appears to be a promising early clinical marker for increased risk of developing OP, and resveratrol metabolic patterns could be a potential therapeutic marker for both resveratrol efficiency and GM composition.

The fact that resveratrol has been clinically shown to have both a regenerative effect on the intestinal mucosa and prebiotic properties is of enormous importance, especially for OP patients, because it can synergistically support conventional standard therapies, such as enhancing calcium absorption in the intestine, and is suggested to modulate side effects such as those of bisphosphonates.

Direct stimulation of anabolic pathways such as Wnt in osteoblasts through modulation of inflammatory signaling cascades such as NF-κB and RANKL also supports the use of resveratrol in OP prophylaxis and in a co-therapeutic approach. It should be emphasized that resveratrol is recommended as part of an integrated therapeutic concept with adequate regular physical exercise, psychosocial stress reduction, and the establishment of anti-inflammatory dietary habits rather than as a monotherapy. Although the metabolism of resveratrol by the GM is still incompletely understood, promising beneficial effects are emerging that support the clinical evidence of improved bone markers and advocate for additional randomized, controlled, clinical trials. A key point to highlight is that natural polyphenols such as resveratrol should be consumed regularly, advocating long-term adaptation and lifelong maintenance as part of an appropriate natural diet.

Education and awareness of the influence of dietary habits on bone metabolism are essential for both physicians and OP patients. In addition, an interprofessional collaboration between basic researchers, gastroenterologists, orthopedic surgeons, trauma surgeons, general practitioners, and endocrinologists should be established for the management of OP patients in order to prophylactically maintain bone health and thus the patient’s quality of life at an early stage and in the long term.

## Abbreviations

ALP	alkaline phosphatase
A.	Achromobacter
*A. equolifaciens*	Adlercreutzia equolifaciens
B.	Bifidobacterium
BMC	bone mineral content
BMD	bone mineral density
BMI	body mass index
BMSCs	bone marrow mesenchymal stem cells
BMP	bone morphogenetic protein
BSP	bone sialoprotein
*C. coccoides*	Clostridium coccoides
CAR	Anti-CD19 chimeric antigen receptor
Col I	Collagen I
COL1A1	alpha-1 type I collagen
CTX	c-terminal telopeptide
DNMTs	DNA methyltransferases
DMSO	Dimethyl sulfoxide
*E. coli*	Escherichia coli
*E. rectale*	Eubacterium rectale
ERs	estrogen receptors
FITC	Fluorescein isothiocyanate
FMP	fermented product
GM	gut microbiota
HDACs	histone deacetylases
IAA	indole-3-acetic acid
IG	immunglobulin
IGF	Insulin-like growth factor
IL	interleukin
lncRNAs	long non-coding RNAs
IS	indoxyl sulfate
KO	knock-out
L.	Lactobacillus
LPS	Lipopolysaccharide
LS-BMD	lumbar spine bone mineral density
MMP	matrix metalloproteinase
MSC	mesenchymal stem cells
NF-κB	nuclear factor-kappa B
NP	nanoparticles
MAPK	mitogen-activated protein kinase
mRNA	messenger RiboNucleic Acid
miRNAs	micro RNAs
NSAIDs	Nonsteroidal anti-inflammatory drugs
OCN	osteocalcin
OP	osteoporosis
OPG	osteoprotegerin
OPN	osteopontin
OVX	ovariectomized
PARP	Poly(adenosine diphosphate [ADP]-ribose) polymerase
PPIs	proton pump inhibitors
RANK	receptor activator of NF-κB
RANKL	receptor activator of NF-κB ligand
ROS	reactive oxygen species
Runx2	Runt-related transcription factor 2
SCFA	short-chain fatty acids
SERMs	selective estrogen receptor modulators
*S. equolifaciens*	Slackia equolifaciens
Sirt	Sirtuin
SOD	superoxide dismutase
spp.	abbreviation for more than one bacteria species
TET	ten-eleven translocation
Th1	Type 1 T helper
Th17	T helper 17
TMAO	Trimethylamine N-oxide
TNBS	2,4,6-trinitrobenzene sulfonic acid
TNF	tumor necrosis factor
TRAP	tartrate-resistant acid phosphatase
TRACP-5b	tartrate-resistant acid phosphatase isoform 5b
Treg	regulatory T cell
uNTx	urinary type I collagen cross-linked N-telopeptide
VEGF	vascular endothelial growth factor
vBMD	volumetric bone mineral content.
Vit.	Vitamin
Wnt	Wingless
WT	wild-type
ZO-1	Zonula occludens-1
